# *In silico* based exploration of natural and synthetic antidiabetic compounds: A comprehensive review of computational approaches

**DOI:** 10.5599/admet.3070

**Published:** 2026-03-06

**Authors:** Ahmad Fariz Maulana, Sriwidodo Sriwidodo, Iman Permana Maksum, Yaya Rukayadi

**Affiliations:** 1 Department of Chemistry, Faculty of Mathematics and Natural Sciences, Universitas Padjadjaran, Sumedang, 45363, Indonesia; 2 Department of Pharmaceutics and Pharmaceutical Technology, Faculty of Pharmacy, Universitas Padjadjaran, 45363, Indonesia; 3 Department of Food Science, Faculty of Food Science and Technology, Universiti Putra Malaysia, 43400 Serdang, Selangor, Malaysia

**Keywords:** ADMET prediction, drug discovery, *in vitro*, *in vivo*, multidisciplinary drug development

## Abstract

**Background and purpose:**

Diabetes mellitus type 2 is a global health issue marked by hyperglycemia and metabolic dysfunction. Despite progress, discovering safe and effective antidiabetic agents remains crucial. This review highlights integrated In Silico, In Vitro, and *in vivo* methods for identifying novel antidiabetic compounds from natural and synthetic origins.

**Experimental approach:**

Computational tools including molecular docking, molecular dynamics, and ADMET prediction identified inhibitors targeting DPP-IV, α-glucosidase, and PPAR. Promising compounds underwent *in vitro* enzymatic and cellular assays, followed by *in vivo* efficacy tests in diabetic animal models assessing glucose levels, biochemical markers, and tissue histopathology.

**Key results:**

Integrated computational and experimental approaches effectively pinpointed compounds with strong target binding, enzyme inhibition, and positive cellular effects. *In vivo* data showed significant glucose reduction, enhanced insulin response, and pancreatic protection. ADMET analysis further supported their drug-likeness and safety profiles.

**Conclusion:**

Combining computational screening with biological validations forms a cost-effective pipeline for antidiabetic drug discovery. Multi-disciplinary integration increases lead identification success, guiding future refinement of *in silico* models and expanded *in vivo* studies to accelerate novel diabetes therapeutic development.

## Introduction

Diabetes mellitus (DM) is a long-term condition that alters the body’s metabolic activities, having all the hallmark features of hyperglycemia. It can, in broad terms, be classified into two categories: type 1 and type 2 diabetes mellitus. The type one variant results from an autoimmune destruction of β-pancreatic cells leading to inadequate insulin secretion. On the other hand, type 2 diabetes is a result of insulin receptor/microcellular clinical resistance and cells in which monosaccharides derived from complex carbohydrates cannot be utilized appropriately. This leads to an excessive buildup of glucose in the bloodstream, marking the onset of hyperglycemia, a symptom of diabetes [1]. Oxidative stress is another metabolic impairment associated with diabetes. Diabetic patients have been shown biochemically to have higher levels of reactive oxygen species (ROS) in their cells and tissues. To combat ROS, there needs to be considerable amounts of potent antioxidants in the patient's body, since antioxidants can limit or completely react with oxidizing substances, thereby preventing the oxidation of other materials [2]. Both forms of diabetes are characterized by difficulties in managing their blood glucose levels. In type 1 diabetes, external insulin administration is a viable option for managing the condition. Simultaneously, for treating type 2 diabetes, different forms of glucose-lowering medications are frequently employed as part of the therapeutic regimen [3].

The prevalence of this disease cannot be considered trivial. The prevalence of DM continues to increase over time. The World Health Organization (WHO) estimates that there will be around 300 million diabetes sufferers worldwide by 2025 [4]. This figure is sufficient to justify the urgent need for new, more effective therapies.

Multiple oral antidiabetic drugs function to either control insulin action alone or alongside insulin. Treatment with sulfonylurea medications can induce insulin secretion, though this effect is often accompanied by an apparent decline in the later stages. This phenomenon, termed “secondary failure”, is believed to result from sulfonylureas’ toxic effects on pancreatic β-cells. Understanding "secondary failure" suggests that β-cell depletion may result from sustained sulfonylurea exposure [5]. The hyperinsulinemia and elevated triglyceride levels associated with obesity can be treated with acarbose, voglibose, and miglitol without promoting weight gain. Acarbose monotherapy or combination therapy is effective; its high price and gastro intestinal GI side effects may inhibit patient adherence. For better tolerance, the first dose should start at 25 to 50 mg *per* day, slowly titrating to 50 to 1000 mg three times a day (TID). In the long term, a regimen of acarbose could foster resistance due to a weakened mechanism. Thus, advancing diabetes treatment systems remains essential to uncover new mechanisms and develop targeted therapeutic strategies [5,6].

The development of antidiabetic drugs entails the identification, design, and testing of new compounds in accordance with new criteria and standards to mitigate symptoms and improve the prognosis of diabetes complications. Various lines of investigation across pharmacology, biology, and technology are being pursued to develop effective, long-lasting solutions for diabetes. The technical plan of concern is directed toward the creation and administration of glucagon and insulin via an artificial pancreas. Despite this, it took ten years for this technique to be finalized and actually utilized. Secondly, researchers in biotechnology have faced challenges relating to the functional pancreatic transplants. Nevertheless, the use of such biotechnologies is severely limited by the geometric similarity of islet cells and the longevity of grafts after heterotopic transplantation. From these studies, it is apparent that pharmacological innovations may be the most realistic and useful for controlling diabetes worldwide [5].

The use of natural products has emerged as one of the focal areas concerning the treatment of many diseases, such as diabetes. The global pharmaceutical market is estimated at around 1.1 trillion US dollars annually. Approximately 35 percent of the global pharmaceutical market comes from natural products such as plants (25 %), microorganisms (13 %), and animals (around 3 %). These figures show how essential natural products are to the global pharmaceutical companies seeking to formulate new drugs. They serve as: direct sources of therapeutic agents or herbal medicines, raw materials in the formulation of complex semi-synthetic drugs, models for key molecular design, as well as taxonomic marks for new drug invention. It is estimated that about one third of the most selling drugs across the globe are either natural products or their derivatives [7].

Exploring natural materials individually would take a great amount of time due to their vast diversity. However, this challenge can be solved using a computational approach. At present, this approach has become an invaluable asset in drug discovery, offering a far more economical and efficient alternative to traditional techniques [8]. A large number of compounds, often running into thousands, may be screened *in silico* in batches to distil a handful down as viable candidates. To illustrate, Halim *et al.* [9], in a 2021 study, virtually screened over 6609 compounds to identify potential α-glucosidase inhibitors. Compounds with such numbers could never be tested in the lab one by one. As such, this method dramatically improves the total number of active compounds that can be screened while minimizing preliminary research costs [9].

This approach saves time and money and is a comprehensive, multi-method approach to implement. Founded on principles of systems theory, an integrative approach entails the use of several methodologies, including molecular docking, virtual screening, quantitative structure-activity relationship (QSAR) modelling, molecular dynamics (MD) simulations, and DFT analysis, which provides complete information about a ligand under study and the corresponding macromolecular complex [10]. Using computer simulations, the interactions between compounds and molecular targets can be predicted with high precision, which can significantly expedite the identification of novel diabetic therapies and elucidate the underlying molecular pathways [11-14]. Such information can subsequently be applied to develop diagnostic and therapeutic strategies [15-17].

The *in silico* method for lead compound identification can be accelerated by computer-aided techniques, yet it still requires biological validation. By reinforcing the connection between in silico methods and laboratory tests (both in vitro and in vivo), it further enhances validation of the *in silico* methods used. For example, molecular docking of the α-glucosidase enzyme showed that the eight test compounds with the best docking scores were validated biologically as potent inhibitors of α-glucosidase activity. [9]. Not only did these compounds serve as inhibitors, but also, the PPAR-γ agonist, benzylidene-2,4-thiazolidinediones, has also demonstrated robust activity *in vivo* [18]. Hence, the *in silico* approach is not merely a theory or simulation; it is linked to experimental evidence and can produce effective drug candidates.

In the early design phases, assessing drug-likeness characteristics is possible with a computational approach. Modern research goes beyond identifying biologically active compounds to include selection based on absorption, distribution, metabolism, excretion and toxicity (ADMET) analysis. These parameters ensure that the test compound is not only active *in silico* but also meets standards for being considered a safe and effective therapeutic drug. In Ali's study [19], candidate glucokinase activators identified through virtual screening were further selected to ensure compliance with Lipinski's rules and favourable synthetic accessibility before being proposed to validate the hypothesis. With this rationale, such an approach increases the likelihood that the selected test compound will be a lead that can be further developed, rather than a random finding.

This article aims to evaluate the impact and role of in silico techniques in discovering antidiabetic compounds from natural and synthetic sources, using methods such as molecular docking, molecular dynamics, QSAR, and virtual screening. This review analyses the existing literature, incorporating the successful application of *in silico* approaches to numerous diabetes-related molecular targets, discusses their relative merits and demerits vis-à-vis experimental case studies, and examines how they combine with *in vitro* and *in vivo* techniques. This review aims to map emerging trends, such as the integration of Artificial Intelligence and big data, to inform strategic decisions on the development of antidiabetic compounds and to highlight opportunities for further research.

## *In silico* principles and methodology in antidiabetic research

### Definition and scope

The growing availability of computer hardware has made computational chemistry an essential tool for drug design, planning, synthesis, and materials science. Methods in computational chemistry, especially those involving quantum chemistry, can be used to predict outcomes that, in turn, can serve as points of reference or for elucidation in a study [20]. The computational approach has the distinct advantage of unlimited resolution compared to experimental approaches that use microscopy. Computational 'microscopy', for instance, renders the motion of atoms in systems of interest at the femtosecond (fs) time scale up to tracking every atom’s movement within the system. This approach provides significant control over the so-called (virtual) laboratory conditions, making reproducibility straightforward, features that are difficult to achieve in standard experimental settings [13].

Such computational experiments can test new theoretical concepts, for instance, the augmentation of intermolecular forces in molecular systems that are too intricate for hand calculation. At the same time, these simulations can estimate the progress of laboratory experiments so that outcomes can be compared. The information obtained from the simulations will yield minimal models of the molecular structure of the experimental findings in the laboratory [21].

In pharmaceutical research, assessing the prospective toxicological danger of a drug candidate is critical in the early stages to save time and resources. Typically, the toxicological danger of a compound is explored using both *in vivo* and *in vitro* techniques. However, as early as the 1970s, *in silico* techniques began to gain popularity for the purpose of predicting drug candidate toxicity [22]. The term *in silico* comes from the word silicium, which is the computer component of silicon; thus, *in silico* methods refer to predictions based on a computational approach.

*In silico* methods involve applying computer simulations and assessments to examine life forms. For antidiabetic research, in silico techniques enable the evaluation of molecular targets associated with diabetes using bioinformatics tools. This includes the computer-aided design (CAD) of potential drug molecules, docking and scoring them with designated targets, and modelling the binding of test substances to their biological targets at the atomic level [23]. Therefore, various *in vitro* compounds can be studied *in silico* first, which would reduce experimental costs and time while decreasing reliance on animal models.

*In silico* techniques offer predictive capabilities based on a compound's structure long before its actual synthesis. This facilitates very early-stage pharmacological screening during drug development, particularly for semi-synthetic compounds, which are often not readily available or present in trace amounts. The reliability of *in silico* methods is critical for their integration into the drug development workflow [22].

The primary focus of *in silico* methods in diabetes research is molecular docking and molecular dynamics simulations. The two techniques differ in their approaches. Molecular docking is used for predicting and estimating the binding of ligands, for example, putative drugs to the active sites of target proteins and the respective binding affinities. In contrast, molecular dynamics simulations can model the time-dependent motion of atoms in macromolecules and of ligands. As indicated by molecular dynamics simulations, it is possible to assess both the stability of a ligand's complex with a macromolecule and the spatial rearrangements that occur over time with respect to the interaction [23]. A combination of these two structure-based methods enables virtual screening of hundreds of test compounds and aids in the rapid identification of the most promising candidates for diabetes therapy. Other applications of *in silico* methods include developing a quantitative structure-activity relationship (QSAR) model that quantifies the correlation between a compound's chemical structure and its biological activity, as well as screening thousands of compounds against a specified target using computer-based virtual screening [24].

*In silico* methods are particularly advantageous for studying molecular targets associated with diabetes mellitus, as they focus on the design of key enzymes and receptors responsible for glucose and insulin signalling pathways. Researchers can simulate the interaction of candidate ligands using three-dimensional structural data of the protein's active site. Molecular docking is a good example of these techniques as it generates multiple ligand conformations and poses with the macromolecule, along with a score indicating the strength of interaction. As such, it is possible to perform *in silico* analyses of hundreds of compounds and only the most promising ones would be selected for *in vitro* or *in vivo* studies [25]. Indeed, prior work has shown that multiple diabetes therapeutic targets are first identified using in silico methods and subsequently validated in preclinical benchmarks [23].

### General methods: molecular docking, pharmacophore modelling, molecular dynamics, ADMET and QSAR

#### Molecular docking

Molecular docking is a computer-based technique that predicts the binding pose, orientation, and binding affinity of a ligand, such as a drug lead, in its associated protein target's active site. It also calculates energy scores to evaluate the stability of the ligand-receptor interactions. In the context of antidiabetic drug development, docking serves as a structure-based in silico virtual screening method to identify potential candidates from enormous compound libraries [26]. If a crystal structure of a diabetes target protein, like DPP-IV or α-glucosidase, is available, thousands of compounds can be virtually docked and subsequently ranked through a binding energy or docking score. This approach is now critical for *in silico* hit identification and optimized lead compound design in the primary phases of drug development [27].

The past thirty years have seen the evolution of molecular docking, driven by structural molecular biology and structure-based drug design. Supported by tools and software for molecular docking, as well as the ability to readily obtain structures of small molecules (ligands) and macromolecules, these tools seek to explain and predict molecular recognition, including estimating possible binding geometries and predicting binding affinity. Docking is usually on small molecules and macromolecules [28].

Docking has many functions and roles in the field of drug development such as in structure-activity relations (SAR), optimization of (bio)macromolecules with their ligands, identification of leads by means of virtual screening, formulation of binding hypotheses to aid in mutagenesis studies, assist in x-ray crystallographic mounting of ligands and electron density capped substrates, dynamic studies of the chemical mechanism, and design of the combinatorial libraries [28].

In the field of antidiabetic drug discovery, molecular docking techniques have effectively pinpointed candidate compounds that interact with critical therapeutic targets. As an example, virtual screening of the membrane receptor TGR5 (GPBAR1) using docking identified new TGR5 agonist candidates predicted to bind more strongly than the native ligand in its crystal structure [27]. In another study, some natural compounds from coffee, such as caffeine and dihydrocaffeate, were predicted by docking to bind strongly to the nuclear receptor PPAR-γ, with binding energies of ΔG ≈ -39.46 and -33.60 kJ mol^-1^, respectively [29]. These docking predictions are consistent with PPAR-γ physiologic role as an antidiabetic target that improves insulin sensitivity. Moreover, docking studies on the enzyme α-glucosidase and its inhibitors showed good agreement with experiment predictions of hydrogen bonding within the binding site, and the position of the ligands met the IV or X-ray crystallography assay results. To illustrate, acarbose, an α-glucosidase inhibitor, when docked to a homology model of yeast α-glucosidase, preserved all the significant interactions described in its crystal structure and docked binding scores of test compounds followed an expected pattern with actual activity. These successes validate that molecular docking accurately predicts binding positions, helps select potential antidiabetic candidates, and provides guidance before the biological evaluation step [30].

In general, molecular docking begins with the preparation of the target protein and ligand in a format supported by the program being used. Target protein preparation begins with removing water and ligands from the macromolecule. Polar hydrogens were added to the macromolecule, and the macromolecule was ready for use. Next, the ligand structure was prepared with its hydrogens. For web-based software (web tools), these two structures can be uploaded to the website at the specified upload location. Offline software, such as AutoDock, has its own protocols [28].

This protocol is consistent with the protocol described in the article by Issa *et al.* [31]. The protein is first downloaded from the database. If the protein is not available, modelling is necessary, either through homology or ab initio modelling. Homology modelling is the fastest option, as it is time-consuming and computationally cost-effective. Next, polar hydrogens were added to the protein structure and minimized. This minimization can be performed using programs such as Chimera, Groningen machine for chemical simulations (GROMACS) or Discovery Studio to ensure that the protein structure is in its most stable energy conformation before being used in the docking stage [32,33]. This process is crucial to ensure that the protein structure has optimal geometry, is free from steric clashes, and represents the biologically relevant natural state of the target protein. The next step was the grid parameter preparation. This involves determining the grid box size and placement, guided by the identification of the protein's binding pocket for the ligand in the literature. This process is important to ensure that the protein structure used has optimal geometry, is free from steric clashes, and represents the natural state of the biologically relevant target proteins. The next step is the preparation of the grid parameters. This preparation involves determining the grid box size, whose placement is based on the results of identifying the protein-binding pocket with the ligand, as reported in [34]. If no such information is available, the grid box can be placed on a potential site predicted using AutoLigand or fpocket, or blind docking can be performed with an optimal box size of 2.9 times the ligand radius of gyration (~2.25 nm) [35].

**Figure 1.**
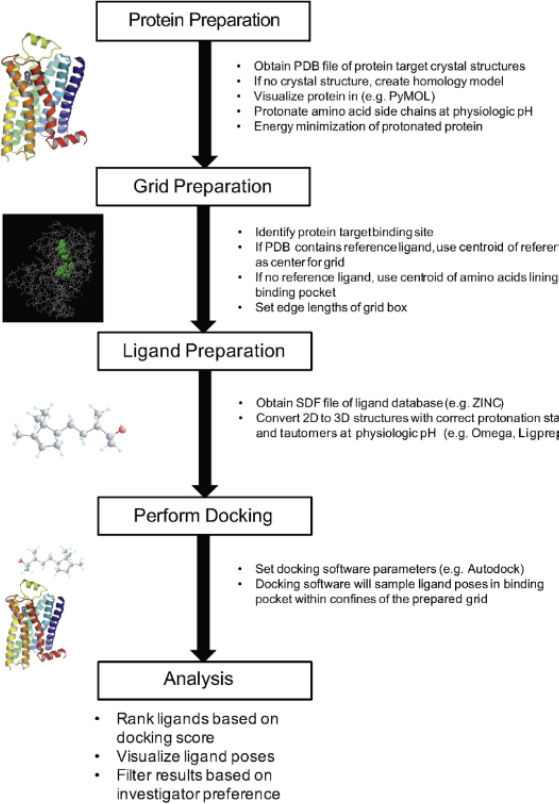
The molecular docking workflow includes protein preparation, grid generation, ligand preparation, docking, and analysis of results to identify the best pose and binding score. Reproduced with permission from Issa *et al.* [31]. Copyright © 2019 Elsevier.

The next step was ligand preparation. Ligands can be obtained from various sources, including the ZINC database. ZINC is a free chemical compound database that provides millions of ligands in 3D format, ready for virtual screening and molecular docking. This database contains commercial compounds, bioactive compounds, fragments, and other classes of molecules, curated and prepared in various protonation and conformational states [36]. ZINC is very useful because it is compatible with many docking software and allows researchers to search, filter, and download ligands based on physicochemical and structural criteria required for computational studies [37]. If a ligand is unavailable, its 2D structure can be constructed and then converted into a 3D structure. Next, the docking parameters were set, and molecular docking was performed. Molecular docking analysis is based on the docking score, RMSD, and ligand position. The docking results are filtered based on user preferences. The molecular docking flowchart by Issa et al. is shown in Figure 1.

In addition to molecular docking, there is ensemble docking. Ensemble docking involves docking ligands against multiple conformations of a target protein, typically generating an ensemble of protein structures to account for conformational flexibility in the structure-based drug discovery. Ensemble docking can be integrated with MD. MD simulations model atomic movements over time using force fields, producing trajectories that capture protein dynamics, including breathing motions and side-chain fluctuations in binding pockets. MD generates diverse protein conformational ensembles by sampling thermally accessible states, which serve as receptor models for docking to better mimic induced-fit binding mechanisms [37-39].

Ensemble docking surpasses traditional single-structure docking, which assumes a static receptor and often misses true binders due to overlooked flexibility. Advantages include higher hit rates (70 to 99 % ligand recovery in G-protein-coupled receptor (GPCR) cases), improved enrichment factors, broader scaffold diversity capture, and reduced false negatives by sampling pocket variations like those from essential dynamics. When integrated with MD, it enhances accuracy for flexible targets without excessive computational cost, as clustering trajectories yields representative structures that capture key variance [40].

Applications shine in challenging systems like G protein-coupled receptors (GPCRs), where MD ensembles identify allosteric modulators and improve virtual screening. This approach is effective for intrinsically disordered proteins (IDPs) in neurodegenerative diseases, kinase inhibitors, and natural product drug discovery, and it predicts binding affinities validated by NMR and long-timescale MD [39]. The following is a protocol for performing ensemble docking integrated with MD simulations.

The procedure begins with preparation of the protein structure using a high-resolution apo or holo crystal structure obtained from the Protein Data Bank [41]. All crystallographic water molecules are removed except those directly involved in ligand binding, followed by the addition of hydrogen atoms, assignment of partial charges, and optimization of side-chain conformations using protein preparation tools such as PDB2PQR or Flare. The protein is then parameterized with an appropriate force field (*e.g.* AMBER ff14SB). For membrane proteins, the system is embedded in a lipid bilayer, then solvated with TIP3P water and neutralized with Na^+^ [42].

Molecular dynamics simulations are subsequently performed to sample protein conformational flexibility. The system is first energy-minimized, then equilibrated under NVT conditions for 100 ps at 310 K, followed by NPT equilibration for an additional 100 ps. Production MD simulations are carried out for 100 to 600 ns using GROMACS or AMBER, with a timestep of 2 to 4 fs, enabled by hydrogen mass repartitioning to improve efficiency. Trajectory frames are saved every 2 to 10 ps, yielding more than 10,000 snapshots. When necessary, enhanced sampling techniques such as metadynamics or accelerated MD are applied to reveal cryptic binding pockets [43].

Representative protein conformations are extracted from MD trajectories using principal component analysis or essential dynamics, focusing on the binding pocket within a 1.0-2.0 nm radius. Trajectories are clustered using RMSD-based methods on backbone or pocket atoms with a cutoff of 0.1-0.2 nm, or alternatively using time-lagged independent component analysis. From these analyses, 5 to 20 cluster centroids are selected, ensuring they capture more than 80 % of the conformational variance. The selected structures are aligned and saved in PDBQT format for subsequent docking [40].

In parallel, the ligand library is prepared by converting all compounds to PDBQT format using OpenBabel or AutoDockTools [44]. Multiple tautomers and protonation states are generated, and partial charges are assigned using the AM1-BCC method to ensure chemical diversity [45]. A docking grid box is defined around the binding site, typically 2.0 to 2.5 nm in size, with an exhaustiveness parameter ranging from 8 to 32.

Ensemble docking is then performed by docking the ligand library against each representative protein conformation using software such as AutoDock Vina, GOLD or DiffDock in a parallelized workflow [46]. Docking results are combined using consensus ranking approaches, such as exponential consensus ranking, and the top-scoring ligands are rescored for each conformation using MM-GBSA calculations or machine-learning-based scoring functions [47].

Finally, the docking results are analysed by clustering ligand poses with RMSD values below 0.2 nm and by calculating average binding scores, interaction fingerprints, and pocket occupancy across the ensemble. Promising ligands are further validated through MD refinement simulations of approximately 50 ns per protein-ligand complex and, where available, compared with experimental data such as NMR chemical shift perturbations. Ligands showing consistent consensus ranking and diverse yet stable binding modes are prioritized for further study [40].

#### Pharmacophore modelling: identifying important chemical features that play a role in biological activity

Pharmacophore modelling is an approach that uses computational methods to discover new drugs and identifies the key features that drive a drug’s biological activity. With pharmacophore models, drug-protein interactions, which are crucial for ligand-based screening and drug development, are better understood. *In silico* screening of potential drug candidates based on pharmacophore models has become a standard practice in drug design. An excellent example is the DPP-IV inhibitor studies, in which lead compounds were discovered through pharmacophore-based virtual screening and subsequent compound-target interaction analysis [48]. Further integration of pharmacophore models with virtual screening through molecular docking, ADME predictions, and molecular dynamics simulations has enhanced their application [49].

The goal of pharmacophore modelling is to seek and apply pharmacophores which are “donors and acceptors of hydrogen bonds, hydrophobic portions, and charged centers” [50]. These principles of pharmacophore understanding are crucial to new drug development because, after the essential feature pattern outline, researchers can find or invent compounds that match those features. Pharmacophore modelling may be either ligand-based, where a set of active synergistic compounds such as antioxidants is known, or structure-based, where the receptors and bound ligands to the interdependencies of crucial interaction elements within the active site of the SARS-CoV-2 target [51].

More recent approaches to pharmacophore modelling have integrated these with molecular dynamics simulations, which can account for the intricate dynamics of macromolecules and their ligands and therefore yield more physiologically relevant interaction patterns. Moreover, the expanding use of machine learning and artificial intelligence in combination with the availability of web servers for 3D pharmacophore modelling is further enhancing Schaller *et al.* [52] findings. With respect to antidiabetic application, pharmacophore modelling can be used to screen compounds or design new ones that mimic specified geometric and chemical milestones of potential candidates [52].

As an example, a three-dimensional pharmacophore model was developed for DPP-IV inhibitors using a study of gliptins, and the database was searched to identify new molecules that resembled the fundamental and skeletal structural chemical features [53,54]. Furthermore, the best candidates identified by docking simulations were shown to bind to the DPP-IV active site and behaved as competitive inhibitors [55]. Also, monograph based pharmacophore design of some herbal compounds was also done for α-glucosidase. Ranade *et al.* conducted a study focused on the discovery of pharmacophore features like two H-donor sites, one H-acceptor site, and an aromatic site, which are shared among quinoline based α- glucosidase inhibitors. This model was then used to develop new derivatives with higher potency to get better results. *In vitro* assay results confirmed that the compound "6c" they designed showed significantly higher α-glucosidase inhibitory activity, with an IC_50_ of approximately 13 μM, compared with acarbose, which had an IC_50_ of 33 μM [56]. This strongly supports the efficiency of the pharmacophore strategy for compound optimization and supports the findings of Ranade *et al.* [56]. It is also noteworthy that active molecule alignment via pharmacophore techniques matched with docking poses at the active site, which suggests that those pharmacophore features were indeed the hot spots for interactions and repose to the binding on the target [30]. To summarize, pharmacophore modelling provides a pivotal framework for developing advanced antidiabetic agents through feature-based virtual screening, which can be complemented by docking and subsequent synthesis of candidate compounds.

There are six main stages in pharmacophore modelling. The first stage is the selection of the training set. Select a structurally diverse set of active and inactive compounds relevant to the biological target. The training set should include molecules with known activity data, ideally spanning several orders of magnitude in potency, and should include both active and inactive molecules to enable model validation and discrimination [57]. The second stage is conformation analysis. In step two, generate multiple low-energy conformations for each molecule in the training set. This step is crucial because bioactive conformation may not be the lowest-energy conformation. Use molecular mechanics or quantum chemical methods to sample conformations, ensuring coverage of relevant conformational space. The third step is identification and extraction. Identify pharmacophoric features (*e.g.* hydrogen bond donors, hydrogen bond acceptors, aromatic rings, hydrophobic centers, ionizable groups) present in active molecules. Feature extraction can be performed using software tools such as Molecular Operating Environment (MOE), LigandScout or Phase [50], which systematically detect and annotate these features in 3D space. The fourth step is molecular alignment and pattern recognition, Align the active molecules based on their pharmacophoric features. Use alignment algorithms to superimpose molecules so that their essential features overlap in 3D space. This step helps to identify common spatial arrangements of features shared among active compounds. The fifth step is pharmacophore model generation. In this step, construct the pharmacophore model by combining the identified features and their spatial relationships. Define inter-feature distances, angles, and tolerances to specify the spatial constraints. The model should be capable of distinguishing active from inactive compounds. And finally, model refinement and validation. Refine the model by adjusting features, constraints, and tolerances to optimize its ability to discriminate between active and inactive compounds. Validate the model using external test sets or cross-validation methods. Metrics such as enrichment factor, ROC curves, and goodness-of-hit (GH) scores are commonly used for validation [57].

#### Molecular dynamics simulation

Molecular dynamics (MD) simulation is part of a computational toolkit that aids in understanding and modelling systems at the molecular level. It enables scientists to investigate the movement and interactions of molecules within a system with respect to physical and chemical processes, as well as to temperature, pressure, and humidity [13].

MD simulations are more advanced than static docking because they place atoms in a protein-ligand system in a more realistic way, moving them according to physical (molecular mechanics) laws over time. MD offers insight into protein mobility and the endurance of the ligand-protein complex in a solvent environment (with solvents, ions, *etc.*) over a nanosecond to microsecond timescale. Using MD, researchers can determine whether the docked pose is stable (the ligand is retained in the binding pocket) or whether drastic conformational changes occur in the protein and ligand. Analysing parameters such as root mean square deviation (RMSD) and root mean square fluctuation (RMSF) shows how the positions of the complex's atoms relative to a reference structure change during the simulation, thereby indicating the complex’s stability. As an illustration, a low plateau feature of the protein RMSD (approximately 0.2 to 0.3 nm) suggests that the protein-ligand structure has attained a steady-state equilibrium, while the remaining RMSF exhibits that the most flexible regions of the protein can be identified [56]. Quantitative evaluation of interaction strength can also be performed by measuring the number of hydrogen bonds formed over time and the binding energy using MM/PBSA or MM/GBSA estimates.

Dynamic simulation of molecules includes modelling molecular shape and energy, along with dynamic calculations that predict changes in a molecule's position and speed over a given time frame. Common methods include particle-based molecular dynamics, molecular dynamics, and Monte Carlo. Each method has its advantages and disadvantages for addressing a given problem and has unique requirements in molecular physics and chemistry [58].

Molecular dynamics simulation results can be used to understand chemical reaction mechanisms, particle motion, and thermodynamic processes; to assess materials and material properties; to optimize processes in design and manufacturing; and to predict the physical and chemical characteristics of final products. There is a growing trend toward the simulation and modelling of molecular dynamics, highlighting its importance across several scientific disciplines [58].

In the process of researching potential antidiabetic compounds, MD simulations validate docking results and elucidate the interaction mechanism at the atomic level. For instance, in a study using TGR5 receptors, Enejoh *et al*. [27] performed MD simulations on a subset of small molecules that had previously been docked to the receptor. These compounds were simulated for 100 ns to assess their stability. The results showed that the leading ligand-TGR5 complex remained stable throughout the simulation, indicating that the ligand was bound to the pocket with firm retention, suggesting high affinity and proper conformation [27]. Similar observations were reported for DPP-IV and α-glucosidase inhibitors, in which docked protein-inhibitor complexes were placed in water and simulated. The complexes displayed almost flat RMSD profiles and conserved key interactions, such as hydrogen bonds with catalytic residues, for example, Glu206/Asp207 in DPP-IV and Asp518/Asp616 in α-glucosidase, which corroborated the strong stability of ligand binding [30,56]. It should be noted that MD simulations in the range of 50 to 100 ns demonstrated the possibility of the new ligand repositioning to form additional interactions not observable in the static structure, providing further avenues for structure refinement. Therefore, MD is a critical in silico validation step in the discovery of antidiabetic compounds: confirming that the candidate binds adequately and interacts with its target in silico before moving on to more expensive biological experiments.

**Figure 2.**
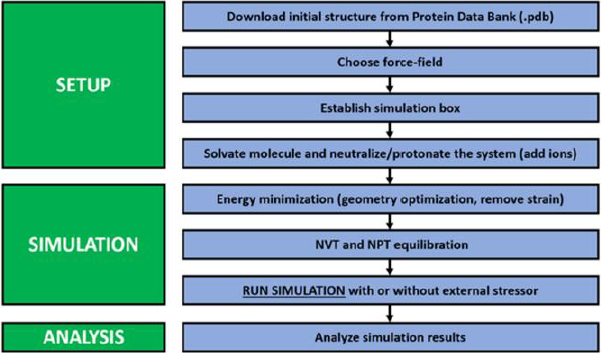
General steps to run a GROMACS MD simulation. Reproduced from Smith *et al.* [60], licensed under CC BY 4.0

Overall, molecular dynamics simulations consist of the initiation, energy minimization, equilibration, production, and trajectory analysis stages, as shown in Figure 2. The initiation process involves preparing the structure, including both macromolecules and ligands, adding ions, adjusting the force field, and providing a water box (for explicit water simulations). In some cases, the simulation system is also prepared by adding a lipid membrane. This is usually done to analyse an antibacterial compound and to study membrane-bound proteins, such as transmembrane and peripheral membrane proteins. The next stage is energy minimization. This stage aims to eliminate bad contacts between atoms from the modelling process, add solvents or place ions, stabilize the initial structure, and prevent structural damage or energy spikes during MD simulation. The equilibration stage consisted of temperature stabilization (NVT) and stabilization of pressure and density (NPT). This stage aims to ensure that the system has reached a stable thermodynamic state and is consistent with the simulation to be performed. The next stage is production. At this stage, thermodynamically representative data can be generated, and the trajectory of the simulation is recorded. This trajectory can then be processed to analyse RMSD, RMSF, radius of gyration, hydrogen-bonding parameters, free energy, and other parameters as required by the researcher [59].

#### ADMET prediction

Besides biological activity, the pharmacokinetics and toxicological aspects of the drug candidate should be analysed at an early phase. The *in silico* methods aim to predict the ADMET of a compound, which stands for absorption, distribution, metabolism, excretion, and toxicity, based upon the chemical structure, to rule out major problematic candidates before actual testing begins in clinical trials. Various drug-like features, including Lipinski’s rule of five [61], which includes molecular weight, log *P* lipophilicity, number of H donors and acceptors, and rotatable bonds, are often used as preliminary tests to evaluate the likelihood of bioavailability [62]. Generally, compounds that violate too many criteria, such as being highly lipophilic or too large, are assumed to have poor absorption or fundamental formulation difficulties, and therefore, are not worth sophisticated further development.

To evaluate the ADMET properties of new molecular entities (NMEs) as early as possible, various *in vitro* and *in vivo* methods, including medium- and high-throughput screening, have been developed, which also facilitate the rapid accumulation of experimental data. However, as the number of NMEs continues to increase, these experimental approaches have several inherent shortcomings: they are time-consuming and costly, and they involve animal welfare issues, which have greatly limited their application and spurred the emergence of *in silico* methods for predicting ADMET properties [63].

As described previously, an ADMET study is a pharmacokinetic evaluation of a drug, where ADMET stands for absorption, distribution, metabolism, excretion, and toxicity. It is a preclinical study that develops predictive models for candidate characteristics, such as how much will be absorbed if administered orally. Or, how much will be absorbed in the body? Both processes are critical to the discovery of new drugs. For example, low absorption will greatly affect distribution and metabolism, potentially leading to serious neurotoxicity or nephrotoxicity. Generally, the purpose of the study is to ascertain the metabolomic disposition of drug molecules within an organism. In that way, ADMET is one of the most important components of the computational drug design process [64].

To assess whether a drug development is good, 3 approaches can be used: Lipinski, Veber, Egan, Pfizer, and GSK. Each regulation has its own provisions, including [65]:

- the Lipinski rule - the rule of the 5 violations (Ro5). The basic rule, according to Lipinski *et al.* [61], states that for four properties, MW ≤ 500 Da, HBD≤ 5, HBA≤ 10 and log *P* ≤ 5. If two properties are outside the domain, poor absorption or permeability is possible, even acceptable.
- The Veber rule, which may be good or not good, states (according to Veber *et al.* [66] ) that the following conditions must be met: rotatable bonds ≤ 10 and tPSA ≤ 1.40 nm^2^ or HBD + HBA ≤ 12
- The Egan rule, or bad/good oral bioavailability rule, which also may be good or not good, stipulates, according to Egan *et al.* [67]. that the following conditions must be met: log*P* ≤ 5.88, TPSA ≤ 1.316 nm^2^
- The GSK 4/400 rule limits the log *P* values of the considered compounds to be less than 4 and the molecular mass (MW) to be less than 400 Da and requires that the generated ADMET profile be favourable: log *P* < 4 and MW < 400 Da.
- Pfizer rule 3/75 specifies that compounds with log *P* > 3 and low tPSA < 75 are about 2.5 times more likely to be toxic than to be clean.

Furthermore, there are numerous models and software such as SwissADME, Pharmacokinetics and cellular Modelling System (pkCMS), and admetSAR [68] that can estimate some ADME parameters such as intestinal permeability (Caco-2), water solubility, plasma protein binding, metabolic enzyme inhibition (CYP450), membrane penetration properties (such as breach of the blood-brain barrier), and acute toxicity or carcinogenic potential. These predictions create an initial profile of the compound concerning how it would interact within the body, regarding the bio-distribution to various organs, the metabolism, and possible toxic ramifications [62].

ADMET Lab 3.0 is a bioinformatics-based web tool designed to estimate the ADMET attributes of a chemical compound. Users can submit a molecular structure, typically in SMILES format, and obtain preliminary forecasts regarding the compound’s pharmacokinetics and toxicity. ADMET Lab 3.0 applies machine learning technology along with a vast database to predict critical factors such as intestinal permeability, oral bioavailability, enzymatic metabolism via CYP450, hepatic toxicity, mutagenicity, and other relevant pharmacokinetics [63].

To screen molecules during the early phases of drug development and identify those with advantageous pharmacokinetic characteristics and a low risk of toxicity, this platform is specifically tailored towards performing these functions. The results of the analysis are presented in a report or table, including both quantitative and qualitative predictions for all ADMET attributes evaluated. This tool is very effective for initial analysis, although it is simple [63]. Static property predictions are more useful for early assessments, as in ADMET Lab 3.0. This makes it better suited for the preliminary evaluation of molecules during the chemical compound screening process, setting it apart from time-based simulation platforms like pkCMS.

The pharmacokinetics and cellular modelling system allows the modelling and simulation of the complete drugs’ lifecycle in the body, including Absorption, Distribution, Metabolism, and Excretion (ADME). pkCMS can predict important parameters such as volume of distribution, clearance, half-life, and bioavailability of a drug using biopharmaceutical information and pharmacokinetic data or molecular structures provided in SMILES notation [69].

In addition to clinical pharmacokinetics and biopharmacokinetics, pkCMS focuses on simulating dose-plasma concentration curves, which depict drug levels in the plasma following a single or multiple doses over time. It assists in deriving optimum dosing and analysing the effect of different treatment strategies [69]. Unlike ADMET Lab 3.0, which is limited to static predictions, pkCMS offers dynamic simulations geared toward comprehensive clinical evaluation, pharmaceutical analysis, and drug development. To conduct an ADMET analysis, researchers simply provide the 2D structure to be analysed into the web tool they will use. This structure is typically in .sdf or SMILES format.

As for paid ADMET analysis software, one of them is ADMETPredictor [70], which is issued by SimulationPlus, Inc. SimulationPlus provides a suite of modelling tools widely used in pharmaceutical and biotechnology R&D to predict ADMET properties, simulate pharmacokinetics, and support model-informed drug development strategies. Its two flagship components, ADMETPredictor and GastroPlus [71], form a connected ecosystem that links early-stage *in silico* property prediction with mechanistic PBPK/PBBM simulations across species and formulations [70].

ADMETPredictor is a machine-learning-based platform that predicts a broad range of ADMET and physicochemical properties, with current versions reporting coverage of more than 100 endpoints relevant to drug discovery and chemical risk assessment. It combines prebuilt models with the ADMET Modeler module, which allows scientists to build custom QSAR/QSPR models from proprietary data using advanced descriptors and algorithms, then deploy these models alongside built-in predictions. Integrated high-throughput PBPK (HT-PBPK) functionality, powered by GastroPlus engines, enables rapid screening of compound series for clearance, exposure, and key PK metrics in seconds rather than hours, which is especially useful for triaging large libraries and focusing experimental resources. In typical workflows, medicinal chemists are able to prioritize structures, flag potential liabilities (*e.g.* solubility, permeability, CYP interactions, DILI risk), and explore optimization directions before synthesis, reducing late-stage failures and accelerating iteration cycles [72].

Owing to the breadth of ADMET assessments, many other programs exist, both free and commercial. Several programs can be used for ADMET analyses. Each program has its advantages and limitations. The following table summarizes some programs for ADMET analysis. Table 1 summarizes some software for ADMET analysis.

**Table 1.** Programs commonly used for ADMET analysis. Some programs are available for free, and some are commercially available

Program/ webserver/ platform	Method	Endpoint	Advantage	Limitation	Ref.
SwissADME	Simple & topological physicochemical descriptors, linear regression models (log *K*_p_), classification models (rule-based & graphical), machine learning (support vector machine), fragmental methods for synthetic accessibility	Bioavailability radar, physicochemical properties, lipophilicity (log*P* o/w), water solubility (log *S*), Pharmacokinetics (Skin permeability (log *K*_p_), HIA & BBB, P-gp substrate, CYP inhibitor), drug-likeness (rule-based & abbot bioavailability score), medicinal chemistry	Combination of multiple models in one tool, fast, free, open and robust model, multiple predictors *per* parameter (+ consensus), competitive in-house model, User-friendly and login-free web interface, Intuitive graphical output (BOILED-Egg, Radar), interoperability in SwissDrugDesign, good performance SA score	Limited scope for novel scaffolds, absence of detailed toxicity profiles, limited customization	[73-75]
pkCSM	Models based on graph-based signatures, distance-based graph signatures, machine learning QSAR models, statistic model based on ADMET/ PK data base	Absorption (Caco-2 permeability, HIA, skin permeability), distribution (volume of distribution (VDss), fraction unbound, BBB permeability), metabolism (CYP450 inhibition (CYP2D6, CYP3A4), substrate likelihood), excretion (Renal clearance), toxicity (hERG inhibition, AMES toxicity, LD_50_, hepatotoxicity)	Comprehensive ADMET prediction in one platform, very easy to use (web-based), more accurate than some rule-based models because it uses graph signatures, quantitative numerical output → suitable for initial screening of many compounds, fast → can process large libraries	Reliance on graph-based signatures, data dependency and accuracy, limited prediction of rare ADMET properties, computational constraints for large datasets	[69,76]
admetSAR	ML models (SVM, RF, kNN, NB), 6 fingerprints, GCN	51 endpoints (solubility, permeability, CYP, toxicity, BBB, hERG, Ames, etc.)	Large dataset; many endpoints; AD present; ADMET optimization present	Database-specific limitations, accuracy for out-of-domain molecules, user interface and updates	[77,78]
ADMETLab	Multi-task deep learning, web-server online	± 119 features: physicochemical, ADME (absorption, distribution, metabolism, excretion), toxicity, medicinal chemistry properties	Wide coverage; accuracy & robustness; batch & API; free & open-access	Reduced accuracy for novel/complex molecule, computational intensity for large-scale predictions, limited algorithm transparency	[63]
QikProp	QSAR models + >40 molecular descriptors (Schrödinger)	log*P*, log*S*, Caco-2, MDCK, logBB, CNS activity, HO absorption, hERG risk, drug-likeness	Accurate for drug-like molecules; fast; integrated with Schrödinger suite; popular in the industry; fast and easy to use; able to comprehensively predict many relevant ADME parameters based on 3D structure; provides a range of values for 95 % of known drugs to compare drug-likeness	Licensing costs as a commercial product, focus on small molecules, dependency on force fields, limited customization and integration	[79,80]
SimulationPlus-ADMET Predictor	ML-based QSAR and neural models	hundreds of ADME/Tox, CYP, hERG, solubility parameters	Comprehensive, accurate, industry-grade	High computational requirements, defined ADMET endpoints, expert interpretation required, cost of licensing	[81]
SimulationPlus-GastroPlus	PBPK mechanistic modelling	Absorption, dissolution, PK profiles, tissue distribution, clearance, DDI	Realistic PK prediction; mechanistic; regulatory use; useful for dose & formulation design	Primary focus on oral absorption, extensive data input requirement, high computational demands: complex PBPK simulations are computationally, steep learning curve	[79]

In the case of antidiabetic drugs, ADMET predictions help select drug candidates that are effective in vitro, safe, and have favourable pharmacokinetic properties. For instance, studies on the antidiabetic flavonoids epicatechin and epiafzelechin from Ficus extracts showed that both met drug-likeness criteria without violating Lipinski’s rules. On the other hand, triterpenes such as lupeol and stigmasterol were considered potential drugs despite violating one of Lipinski's criteria (log *P* > 5) [62]. Such information is important because no matter how effective an α-glucosidase inhibitor is, its possible poor absorption or organ toxicity severely limits its usefulness.

That is why most *in silico* studies are over ADMET screening for antidiabetic compounds. One review noted that, on average, plant-derived antidiabetic agents are evaluated for drug-likeness and ADMET before docking, followed by molecular dynamics simulations [82]. This tiered approach helps ensure a balance between efficacy and important pharmacokinetic attributes for candidates advancing to biological testing. This indicates that early ADMET predictions improve the likelihood of successfully developing effective and safe antidiabetic agents.

#### Quantitative structure-activity relationship

QSAR is an example of mathematical modelling that establishes a quantitative relationship between a molecule's structure and its biological activity. The assumption here is that any changes to structure (e.g., physicochemical properties or molecular descriptors) will affect biological activity. In the QSAR model, every compound is represented with numerical descriptors such as log *P*, atomic charge, molecular volume, number of aromatic rings, *etc*. statistic or machine learning techniques are employed to determine an equation or algorithm that would estimate the activity (IC_50_ value of enzyme inhibition, agonist potency, or other pharmacological parameters) of a new compound based on these descriptors [83]. The importance of QSAR lies in its ability to enable researchers to *in silico* screen and rank large numbers of molecules, offering a deeper understanding of which structural features increase or decrease activity, thereby guiding modifications aimed at optimizing candidate drugs.

To perform quantitative structure-activity relationship (QSAR) modelling, begin by curating a diverse dataset of at least 30-50 compounds with experimentally measured activities (*e.g.* pIC_50_ values spanning several orders of magnitude), standardizing structures through removal of salts and solvents, charge neutralization, tautomer canonicalization, and duplicate elimination using InChI keys, while filtering out inorganics, organometallics, and mixtures; verify structural integrity with identifier consistency checks and outlier detection via principal component analysis (PCA) on descriptors. Next, calculate a comprehensive pool of 500 to 2000 molecular descriptors, including constitutional, topological, geometrical, electronic, and quantum-chemical features, using software like Dragon or PaDEL [84,85], then preprocess by eliminating constants, near-constants, and highly correlated pairs (*r* > 0.9) to yield 100-300 informative descriptors and mitigate multicollinearity. Proceed to split the dataset rationally into training (70 to 80 %), validation (10 to 15 %), and external test (10 to 15 %) sets employing sphere exclusion or Kennard-Stone algorithms, ensuring representativeness in descriptor space as confirmed by PCA score plots to prevent overfitting. Build models through variable selection with genetic algorithms, stepwise regression, or partial least squares to select 4 to 8 optimal descriptors, applying multiple linear regression (MLR), support vector machines (SVM), or random forests while prioritizing balanced complexity (*e.g.* VIF <5) via combinatorial protocols. Validate rigorously by assessing internal predictivity with leave-one-out cross-validation (*Q*^2^_LOO >0.5), bootstrapping (*Q*^2^_BOOT >> 0.5 over 5000 iterations) and Y-randomization, followed by external evaluation using *R*^2^_test > 0.6 and RMSE_test; define the Applicability Domain (AD) *via* Williams plots (warning leverage h* = 3(*p+1)*/*n*, residuals > 3*σ*), where *p* is the number of model variables (descriptors plus an intercept) and *n* is the number of training compounds and *σ* is the standard deviation of the training-set residuals, to identify reliable predictions. Finally, interpret key descriptors for mechanistic insights (*e.g.* positive coefficients indicating favorable electronic effects), deploy consensus models from top performers for virtual screening within the AD, integrate with docking or ADMET predictions for hit prioritization, and iterate with new data for ongoing refinement [86,87].

However, the utility and credibility of any QSAR model are entirely dependent on its validation [88,89]. The validation process is a critical checkpoint to ensure that a developed model is statistically robust, internally consistent, and possesses genuine predictive power for new, unseen chemical entities. Without rigorous validation, a QSAR model may be "overfitted”, meaning it has memorized the training data's noise and specificities rather than learning the underlying structure-activity relationship, rendering its predictions for new compounds unreliable [89,90]. This could lead to erroneous conclusions, misdirected synthesis efforts, and wasted resources in drug development programs. The Organisation for Economic Co-operation and Development (OECD) has established principles for QSAR modelling that underscore the central role of validation in establishing a model's scientific reliability for a specific purpose [91].

Internal validation methods are designed to assess the robustness, stability, and internal consistency of a QSAR model by utilizing the same dataset from which it was constructed. The primary objective is to diagnose potential overfitting and confirm that the model has captured a generalizable relationship between structure and activity [89,91].

To ensure the robustness, reliability, and predictive validity of the developed QSAR model, several internal validation strategies were systematically applied. These validation approaches are essential for assessing model stability, preventing overfitting, and verifying that observed predictive performance is not driven by chance correlations. A cornerstone of internal validation is cross-validation, in which the dataset is systematically partitioned. The most common forms are leave-one-out (LOO) and k-fold cross-validation [89,91].

1. Leave-one-out (LOO) cross-validation: In this procedure, each compound in the dataset is sequentially excluded one at a time, and a model is built using the remaining compounds. The activity of the single excluded compound is then predicted by this new model. This process is repeated until every compound has served as the validation point. While computationally intensive for large datasets, LOO provides a thorough test of the model's internal predictive ability
2. k-fold cross-validation: To mitigate the computational load of LOO, the dataset is randomly divided into 'k' subsets or folds. The model is then trained using 'k-1' folds and validated on the single remaining fold. This is repeated 'k' times, ensuring each fold serves as the validation set exactly once. A 5-fold cross-validation, for example, involves partitioning the data into five sets, with four used for training and one for testing in each iteration [92]. Similarly, a 10×10 % internal cross-validation was used in a study to predict phospholipidosis, in which 10 % of compounds were iteratively held out for testing [93]. The results from all 'k' iterations are then averaged to provide a single performance estimation.
3. y-randomization (response permutation): This technique serves as a crucial check against chance correlations. The process involves rebuilding QSAR models multiple times, each using the original molecular descriptors but with the training set's biological activities (the y-variable) randomly shuffled. A robust model should show significantly lower predictive performance (*e.g.* low *R*^2^ and *Q*^2^ values) on these randomized datasets compared to the original model. If the randomized models perform well, it suggests that the original model's performance may be due to a chance correlation rather than a true structure-activity relationship, and the model should be discarded as unreliable [89].

The primary metric derived from cross-validation is the cross-validated coefficient of determination (*Q*^2^ or *q*^2^). A *Q*^2^ value greater than 0.5 is generally considered indicative of a model with significant predictive ability. Other statistical parameters, such as the standard error of prediction, are also evaluated [91,94].

External validation is universally regarded as the most stringent and conclusive test of a QSAR model's predictive capability and its ability to generalize to new chemical entities [89,91,93]. This method assesses the model's performance on an independent dataset, often referred to as a test set, that was excluded from all stages of model construction and internal validation [92,93].

The process involves initially splitting the total available data into a larger training set (typically 70 to 80 %) and a smaller external test set. The QSAR model is built and optimized exclusively using the training set. Subsequently, this finalized model is used to predict the biological activities of the compounds in the external test set. The model's predictive power is then quantified by comparing the predicted values with the experimentally observed values for the test set [89].

Several metrics are used to evaluate external validation performance. For regression models, these include the predictive *R*^2^ (*R*_ext_^2^ or *r*_pred_^2^) and the correlation coefficient (*R*) between predicted and observed activities [89]. For classification models, common metrics include sensitivity (true positive rate), specificity (true negative rate), overall accuracy, balanced accuracy (BACC), and the area under the receiver operating characteristic curve (AUC) [90,92]. For instance, validation of ADME QSAR models on a test set of marketed drugs yielded balanced accuracies ranging from 71 to 85 %. Another study validating a 3D-QSAR server reported an external *R*^2^ of 0.934, sensitivity of 86.9 %, specificity of 94.5 % and an AUC of 0.981, indicating strong predictive performance. Successful external validation provides strong evidence that the model is suitable for real-world applications, such as screening large chemical databases for potential antidiabetic drug candidates [90,92].

The applicability domain of a QSAR model is the theoretical region within the chemical space for which the model is deemed reliable to make predictions [89]. This space is defined by the structural, physicochemical, and/or biological characteristics of the compounds used in the training set. Predicting the activity of a compound outside this domain is considered extrapolation, which can lead to unreliable or inaccurate results [89,93]. Establishing a well-defined AD is therefore essential for the responsible and credible use of QSAR models, ensuring that predictions are made only for compounds that are sufficiently similar to those the model has learned from [92]. In regulatory or drug-development contexts, a clearly defined AD is a prerequisite for model acceptance.

Several methods are used to define a model's AD described in Table 2. These approaches can be broadly categorized into descriptor-based, geometric, and distance-based methods.

**Table 2.** Overview of major applicability domain (AD) method categories used in QSAR/QSPR modelling, including their conceptual basis and representative techniques for defining model reliability and prediction confidence

AD method category	Description	Specific techniques
Leverage/influence	Identifies compounds that are outliers in the descriptor space and have a high influence on the model	Leverage approach, Williams plot
Distance-based	Defines the AD based on a compound's similarity or distance to the training set compounds.	Euclidean distance, Mahalanobis distance, similarity indices
Fragment/substructure-based	Determines applicability based on the presence or absence of specific chemical fragments found in the training set	Fragment representation analysis (*e.g.* in MC4PC)
Probability-based	Uses the probability of a compound belonging to a certain activity class as a measure of confidence	Probability of membership (*e.g.* in MDL-QSAR)

Table 2 summarizes the main categories of methods used to define the applicability domain of a QSAR model. Leverage-based methods focus on the statistical influence of each compound, with the leverage value indicating how far a compound's descriptor values deviate from the training set average. The Williams plot provides a convenient visual tool for this analysis. Distance-based methods use distance measures in the multidimensional descriptor space to quantify similarity. Fragment-based approaches are more structural, ensuring that a new compound contains substructures that the model has previously encountered. Finally, probability-based methods, often used in classification models, provide a confidence score for a prediction, which can be used to define the AD.

The ​**​**leverage approach​​ quantifies the influence of each compound on the regression model. A compound with a high leverage value is structurally distinct from the bulk of the training set and has a greater impact on the model's parameters. The leverage threshold is often set at 3*p*/*n* [89].

The Williams plot is a graphical tool that visualizes the AD by plotting standardized residuals (prediction errors) on the y-axis against leverage values on the x-axis for all compounds (both training and test sets). Horizontal lines are typically drawn at standardized residuals of ±3, and a vertical line is drawn at the critical leverage value. Compounds that fall within this defined rectangular area are considered to be within the AD and are predicted reliably. Compounds with high leverage are outside the AD, while those with large residuals but low leverage are outliers within the AD [89].

Distance-based methods define the AD by measuring the similarity of a new compound to the compounds in the training set. A compound is considered within the AD if its distance to the training set (*e.g.* to its nearest neighbor or the centroid of the set) is below a defined threshold [92,93]:

1. Mahalanobis distance: This is a powerful metric that measures the distance between a point and a distribution, considering the correlations between the variables (descriptors). It is used to assess how similar a new compound is to the multivariate distribution of the training set compounds
2. Fragment-based similarity: Some models, like the MC4PC program, define the AD based on substructural representation. A compound might be considered outside the domain if it contains a certain number of atom fragments that were not present in any of the training set molecules
3. Chemical space analysis: This involves projecting the high-dimensional descriptor space into a lower dimension (*e.g.* using principal component analysis (PCA)) to visualize the distribution of training and test compounds. If test compounds fall within the area occupied by the training set, they are considered to be within the AD.

The principles of validation and AD are universally applicable but have specific nuances when applied to different QSAR methodologies like Machine Learning-based QSAR (ML-QSAR) and three-dimensional QSAR (3D-QSAR).

ML-QSAR employs a wide array of sophisticated algorithms, including artificial neural networks (ANN), support vector machines (SVM), random forests (RF) and, more recently, graph convolutional neural networks (GCNNs) [95].

The inherent complexity and flexibility of ML models make them highly prone to overfitting. Therefore, rigorous internal validation (*e.g.* k-fold cross-validation) is crucial not just for performance assessment but also for tuning model hyperparameters. External validation remains the ultimate test of generalization [92].

Defining the AD is especially critical for ML-QSAR models, which can sometimes function as "black boxes." The AD provides a necessary boundary to ensure that these powerful algorithms are not applied to compounds for which their predictions would be unreliable. A phenomenon known as "activity cliffs", where structurally similar molecules exhibit large differences in activity, can severely affect the generalization ability of ML models, highlighting the need for careful AD analysis that considers local, as well as global, similarity [89,94].

3D-QSAR methods, such as comparative molecular field analysis (CoMFA) and comparative molecular similarity indices analysis (CoMSIA), correlate biological activity with molecules' 3D properties, including steric and electrostatic fields. Validation in 3D-QSAR follows the same internal and external principles but explicitly assesses the model's ability to predict activity from 3D molecular features. For antidiabetic targets, where specific ligand-receptor interactions are key, 3D-QSAR models are often built using docked ligand conformations, and their validation confirms the predictive power for these spatial interactions. The AD for 3D-QSAR is more complex as it must account for molecular alignment and conformational flexibility. A new compound must be similar to the training set, not just in its 2D structure but also in its ability to adopt a relevant 3D conformation and align properly within the defined field. The interpretation of 3D-QSAR models often involves visualizing contour maps that show favorable and unfavorable regions for specific properties around molecules. These maps provide an intuitive, visual representation of the model's SAR and implicitly help define the AD for structural modifications [90,91,94]

In the development of antidiabetic drugs, QSAR methodologies are systematically used to analyse the structure-activity relationships (SAR) of compounds designed to target critical enzymes or receptors. Several studies have examined the derivatives of the enzyme DPP-IV and its counterpart, the α-glucosidase inhibitor. Dozens of synthetic and natural α-glucosidase inhibitors were tested, and activity data were used to develop both 2D and 3D QSAR models. Recently, a study of 66 quinoline derivatives identified as α-glucosidase inhibitors employed an atom-based 3D QSAR method that developed a model with extremely high *R*^2^ values (*R*^2^ ≈ 0.96 and *Q*^2^ ≈ 0.92 for prediction). This model and its predictions were successfully elucidated with features that enhance activity, including the presence of certain substituents, which may increase hydrogen or other hydrophobic interactions with the enzyme and even predicted the activities of new compounds prior to their synthesis. Indeed, several designed compounds were synthesized and tested, and the measured inhibitory activities confirmed alignment with the QSAR predictions, demonstrating the model's reliability [56]. QSAR has also been used to design high-efficacy, low-side-effect novel analogues of PPARγ agonists. The model was built based on hundreds of thiazolidinedione (TZD) derivatives and PPARγ-active herbal compounds. It was shown that certain substitutions within the hydrophobic ring could preserve agonistic activity while reducing lipophilic side chains [96,97]. In general, the effectiveness of QSAR is marked by its ability to estimate the efficacy of new compounds predicted to function as enzymes, DPP-IV inhibitors, or insulin receptor modulators, thereby prioritizing the synthesis and testing of only those molecules with high predicted activity. In addition, QSAR can be extended to estimate parameters such as ADMET (sometimes called QSAR (Q)SAR when including toxicity) [72], making it a flexible approach for the purpose-driven design of antidiabetic compounds. QSAR, in conjunction with the ever-growing volume of cross-disciplinary biological and chemical information, is paired with contemporary machine learning algorithms to enhance prediction reliability. This integration facilitates the design of ideal lead molecules for therapeutic interventions in diabetes management.

To support *in silico* studies in antidiabetic drug discovery, a wide range of computational software has been developed, each differing in methodological approach, application scope, strengths, and limitations. These tools collectively cover key stages of computer-aided drug design, including molecular docking, molecular dynamics simulations, QSAR modeling, and protein structure prediction. A comparative overview of representative software packages, along with their underlying methods, typical applications, advantages, and inherent limitations, is presented in Table 3. This comparison highlights that the selection of an appropriate computational tool depends on the specific research objective, the target system's complexity, and the available computational resources.

**Table 3.** Representative computational software commonly used in *in silico* drug discovery studies beyond ADMET prediction, highlighting their methodological approaches, typical applications, key advantages, and limitations

Software	Methods	Output	Advantages	Limitation	Ref.
Autodock/ AutoDock Vina	Molecular docking (rigid docking; semi-flexible (Vina))	Binding pose prediction and affinity estimation (scoring)	Free, light computational cost, widely used, lots of supporting literature	Rigid/semi-rigid proteins — do not capture full flexibility; simple scoring	[98,99]
Glide (Schrödinger)	Molecular docking	Binding pose, docking score (HTVS/SP/XP), covalent docking	High precision	Commercial (premium); need more computational cost than Vina; more complex	[100]
MOE	Docking, QSAR, pharmacophore, cheminformatics	Docking, protein modelling, pharmacophore, QSAR, bioinformatics analysis	Versatile and integrated computational chemistry platform	UI is less intuitive; outdated UI; complex features; commercial software with licensing costs; may require significant training	[95]
AMBER	all-atom md (explicit solvent. enhanced sampling)	Trajectory dynamics, conformational sampling, free energy methods	Validated force fields, GPU acceleration, enhanced sampling techniques	High computational cost; complex setup; theoretical model that requires experimental validation	[101,102]
NAMD	MD simulation, scalable parallel MD	Classical MD, enhanced sampling, free energy, QM/MM, very large system MD	Highly scalable; GPU-resident 2× faster; support for systems >100 million atoms; large force field; VMD integration	Low modularity; communication-heavy PME; GPU-PME scaling ≤4 nodes; GPU idle on legacy schemes; small systems less efficient	[103]
GROMACS	Molecular dynamic simulations	Trajectory generation, conformational and energetic dynamics analysis	Free and open-source; highly optimized and fast; supports multiple force fields	It requires a large amount of computing resources due to the highly complicated molecular structure; the accuracy of the force field is the main factor that affects the reliability of GROMACS molecular dynamics simulation results	[104]
AutoQSAR	Automated ML-QSAR builder	Automatic descriptors, ML model generation, validation, ranking score	Fully automated; reproducible; faster; no expertise required; models often outperform manual; rigorous validation	Limited interpretability; not optimal for small datasets; limited user control; sensitive to data quality; descriptors only from internal set	[105]
SwissModel	Homology modelling, template search using BLAST and HHBlits	Predicting structures for proteins with detectable homologs, generating models for docking and virtual screening, mapping variants or mutations, and exploring quaternary structure when oligomeric templates exist	Fully automated, web-based and free, integrated template library, quality estimation (GMQE, QMEAN, QSQE) provide a coherent workflow from sequence to validated model, and benchmarking shows performance among the top servers in blind assessments such as CAMEO, supports both tertiary and quaternary structure modelling using curated oligomeric templates, which is useful when studying complexes and assemblies	depends on the availability and quality of suitable templates; the rigid-fragment assembly and automated pipeline may struggle with large insertions, compared with expert-guided modelling or more flexible packages (for example, MODELLER or hybrid AI/physics approaches), it offers less control over modelling parameters and sometimes fails to produce models for problematic targets	[106]
MODELLER	Homology modelling	multi-template modelling, loop refinement, modelling of insertions/deletions, and integration with other tools (*e.g. via* Chimera interfaces) in structural biology pipelines	allowing systematic combination of homology-derived and stereochemical restraints, flexible and scriptable, efficient and free for academic use	Strong dependence on alignment and template quality, limited ability to handle large conformational changes, long flexible loops, or very low-identity targets.	[107]

### Molecular targets in diabetes treatment

#### Dipeptidyl peptidase-4

Dipeptidyl peptidase-4 (DPP-IV) is a transmembrane protease enzyme responsible for the inactivation of incretin hormones, GLP-1 and GIP, by removing two amino acids from their N-terminal ends. Because GLP-1 and GIP are designed to stimulate insulin secretion, suppress the release of glucagon and enhance insulin release while neutralizing glucagon, DPP-IV’s actions will diminish the stimulation of insulin and augment the release of glucagon, with the net effect of impairing blood glucose control. Inhibition of DPP-IV leads to sustained incretin action, which increases insulin secretion and decreases glucagon secretion. This has proven to clinically normalize glucose levels. This treatment paradigm has proven effective clinically with the development of gliptin (DPP-IV inhibitors) drugs that are extensively used for treating Type 2 diabetes. *In silico*, DPP-IV's known structure (several crystal structures of DPP-IV-inhibitor complexes are available) enables evaluation of ligand interactions through molecular docking and simulation. For instance, hemorphin peptide docking and molecular dynamics simulations showed that the peptide binds to conserved active-site positions in DPP-IV, thereby accounting for its inhibition. These medications help manage postprandial hyperglycemia by reducing carbohydrate bioavailability [108]. Consistent with this goal, numerous *in silico* studies have been conducted to identify new and more potent α-glucosidase inhibitors, including those derived from natural sources. As an illustration, virtual screening followed by molecular dynamics simulations of compounds derived from the plant *Calotropis procera* revealed several highly accessible α-glucosidase inhibitor candidates, including taraxasterol.

#### α-glucosidase

α-glucosidase is a carbohydrate-active enzyme (CAZy family GH31) found on the brush border membrane of enterocytes, which acts as an exopeptidase to hydrolyse starches and disaccharides into glucose monomers to be transported in the blood. Its role in augmenting the rise of postprandial blood glucose is significant. Thus, inhibiting α-glucosidase slows carbohydrate digestion and absorption, blunting the postprandial surge in plasma glucose levels [109]. Therapeutic approaches that incorporate α-glucosidase inhibition have led to the development and clinical use of three oral α-glucosidase inhibitors for type 2 diabetes: acarbose, voglibose, and miglitol [110]. Correspondingly, numerous in silico studies aim to develop more potent, selective α-glucosidase inhibitors, including those of natural origin. One example is the Calotropis procera plant, which underwent virtual screening and molecular dynamics simulations, identifying several novel candidates, such as taraxasterol, with high binding affinities for α-glucosidase inhibitors. These compounds demonstrated reduced binding energy (i.e., increased binding strength) compared with the reference ligand and interacted with key residues in the enzyme's active site, supporting their hypothesis that they are potential antidiabetic α-glucosidase inhibitors that require further validation *in vitro* and *in vivo* [109].

#### Peroxisome proliferator-activated receptor gamma

Peroxisome proliferator-activated receptor gamma (PPARγ) is a nuclear receptor predominantly found in adipose tissue and muscle and is known to be expressed in macrophages. PPARγ is responsible for transcriptional regulation of genes involved in the differentiation of adipocytes, metabolism of fatty acids and insulin sensitivity. PPARγ activation through agonist binding leads to the modulation of insulin sensitivity in peripheral tissues by augmenting the storage of fatty acids in adipose tissue, and modulating adipokines which ultimately decrease blood glucose levels. This makes PPARγ one of the best candidates for targeted therapy in type 2 diabetes; it is treated with TPZ drugs (thiazolidinediones, TZDs) such as rosiglitazone and pioglitazone, which enhance glucose uptake in muscle cells and suppress its production in the liver. However, full PPARγ agonists are potent but also carry strong side effects, such as edema and weight gain, due to overactivation of adipogenesis pathways. This has led to recent research on partial PPARγ agonists and selective modulators that induce insulin sensitization without major side effects. These PPARγ modulators have been identified using computational approaches to drug design based on their structures [111].

For example, one candidate identified through virtual screening of natural compound libraries is podophyllotoxone, which acts as a partial PPARγ agonist. Podophyllotoxone is known to bind to the PPARγ ligand binding domain and displays partial submaximal agonist activity in TR-FRET assays. Lian *et al*. [111] describe this subset of findings. The *in silico* and *in vitro* findings support considering podophyllotoxone a strong lead candidate for further development as an antidiabetic agent, with a proposed mechanism of action involving partial modulation of PPARγ activity.

#### AMP-activated protein kinase

AMP-activated protein kinase (AMPK) is a heterotrimeric kinase that functions as an energy sensor for the cell. AMPK is activated when the AMP/ATP ratio increases (indicating low energy). In response, cellular systems that conserve energy and enhance ATP production get activated. Activated AMPK increases glucose uptake in skeletal muscle by translocating GLUT4, increases lipid (fatty acid) oxidation in adipose (fat) tissue and liver, and decreases gluconeogenesis in the liver. These effects increase insulin sensitivity and greatly improve glucose homeostasis, making AMPK a strong candidate for targeting in the treatment of diabetes and metabolic syndrome. There is evidence that AMPK regulation is dysfunctional in animals and people with diabetes and metabolic disorders; however, AMPK activation (via physiological or pharmacological means) improves insulin sensitivity and metabolic health. It is known that some existing antidiabetic therapies act (partly) through the AMPK pathway; for instance, metformin, which acts indirectly through AMPK in the liver, and PPARγ agonists (TZDs), which can increase the activity of AMPK in the target tissues [112].

In the quest for increasingly specific AMPK activators, *in silico* research has focused on the enzyme's allosteric regions. For example, a study employing structure-based virtual screening identified several novel small molecules that bind to and potentially activate the AMPK regulatory subunit (β1 isoform). The screening yielded 12 candidate compounds for AMPK β1 activators, with diverse scaffolds, that may be further refined as antidiabetic agents that enhance AMPK signalling [113].

#### Glucose transporter type 4

The most prominent isoform of glucose transporter stimulated by insulin is glucose transporter type 4 (GLUT4), which is found mainly in skeletal muscle and adipose tissue. GLUT4 is maintained in a basal intracellular vesicle storage system, but insulin stimulation activates a phosphorylation pathway (IRS-1-PI3K-Akt) that promotes GLUT4 vesicle translocation to the plasma membrane, thus enhancing glucose uptake from blood into cells. This transport process is critical for metabolic glucose homeostasis, particularly postprandially, and failure to properly signal insulin or translocate GLUT4 would impair glucose uptake by muscle or adipose tissues. Reduced GLUT4 translocation is a hallmark of insulin resistance and type 2 diabetes, leading to elevated blood glucose levels due to impaired peripheral tissue uptake [114]. On the other hand, augmentation of GLUT4 translocation to the membrane improves its uptake and insulin sensitivity (insulin, exercise, or certain drugs). This enhanced action of GLUT4 is the main objective of numerous anti-diabetic strategies [115].

Within the *in silico* framework, GLUT4 is frequently identified as an endpoint of the pathway, although it is not an enzyme readily modulated by small ligands. Some studies have attempted to model the effects of compounds on GLUT4 expression or transport using network models or molecular docking of regulatory proteins. GLUT4 translocation to muscle and fat cell membranes is enhanced by the alkaloid alloperine through PKC and PI3K/Akt pathway activation. Although ammonium alloperine’s action is complex, its use is justified by predictive modelling approaches, including network pharmacology and molecular docking of upstream receptors, which have, albeit indirectly, suggested alloperine actions that enhance GLUT4 expression and insulin sensitivity [115].

#### Glucokinase

Glucokinase (hexokinase IV) is a pivotal enzyme involved in cellular glucose sensing located in the liver hepatocytes and pancreatic β-cells. Compared to other hexokinase isoforms, Glucokinase (GK) demonstrates a high Km value of ~8 mM and is not inhibited by the byproduct of its reaction, glucose-6-phosphate. Therefore, GK’s activity is directly proportional to glucose levels above a certain threshold, thereby aiding the determination of the glucose concentration necessary for insulin secretion. In β-cells, GK facilitates the conversion of glucose to G6P, the rate-limiting step in the insulin secretion pathway, whereas in the liver, GK increases glycogen synthesis postprandially. The significance of glucokinase in maintaining glucose homeostasis, particularly in the context of specific genetic conditions, is remarkable. For example, loss-of-function mutations lead to persistent hyperglycemia in individuals with Maturity Onset Diabetes of the Young type 2 (MODY2), whereas gain-of-function mutations cause hypoglycemia due to increased insulin secretion [116]. Consequently, there is an ongoing investigation into the use of Glucokinase Activators (GKAs) as therapies for type 2 diabetes to reduce the threshold for insulin release. Through small GK activators, modulation of the enzyme should restore physiological glycemia; the challenge is to circumvent excessive insulin-triggered hypoglycemia.

GKAs have been classified into distinct classes and developed from both natural and synthetic compounds tailored to these classes through drug design efforts. For some analyses, especially those using docking approaches, the spatial structures of GK in both the free and activator-bound forms are available, which greatly facilitates the use of computational methods. For instance, a candidate GKA, mangiferin, was identified through structure-based virtual screening of thousands of compounds, a form of automated docking. Complementary experiments demonstrated that mangiferin activated GK *in vitro*, and in diabetic mice bearing the db/db mutation, reduced glucose levels without inducing dangerous hypoglycemia [117]. This supports the effectiveness and safety of GKAs derived from mangiferin, demonstrating its potential as a new framework for antidiabetic drug development.

#### Protein tyrosine phosphatase 1B

Protein tyrosine phosphatase 1B (PTP1B) is a non-receptor tyrosine phosphatase that exerts an intracellular action terminating the insulin signalling cascade. PTP1B will turn off the receptor tyrosine kinases (RTK) by dephosphorylating tyrosines on the IR (insulin receptor) as well as on its major substrates IRS-1/2 (insulin receptor substrates 1 and 2). In simpler terms, the PTP1B functions as a negative “brake” for the insulin signalling cascade. In cases of insulin resistance or type 2 diabetes, current models suggest that both PTP1B expression and activity increase, thereby reducing IR/IRS phosphorylation and attenuating insulin action. These findings have been corroborated by animal studies. The absence of PTP1B results in increased peripheral insulin sensitivity and resistance to diabetes; excess PTP1B leads to glucose intolerance. From this, it follows that PTP1B inhibitors are attractive as an adjunct therapy to enhance insulin action. PTP1B inhibition would preserve tyrosine phosphorylation of IR/IRS, thereby increasing glucose uptake into cells and reducing blood glucose. Such an approach may also enhance leptin signalling (PTP1B also modifies the leptin receptor), providing dual action in obese diabetic patients. To date, drug discovery has identified several candidates, including PTP1B inhibitors such as derivatives of aromatic carboxylic acids, diphenyls, benzofurans, and certain natural polyphenolic compounds [118].

The selectivity of PTP1B inhibitors to avoid disrupting other phosphatases, as well as their cell permeability, are barriers to PTP1B clinical development. However, there remains some continuity with *in silico* approaches to compound optimization. To illustrate, one study reported the extraction of Fudan-Yueyang *Ganoderma lucidum* (FYGL), a high-molecular-weight proteoglycan from the mushroom *Ganoderma lucidum* and characterized it as a potent PTP1B inhibitor. Molecular docking assessments conducted demonstrated competitive binding of FYGL to the active site of PTP1B. Furthermore, administration of FYGL in vivo to ob/ob mice resulted in reductions in glucose and body weight, improved insulin resistance, and increased IRS-1/AKT phosphorylation in muscle tissue. These observations confirm that PTP1B is a target of interest and support the notion that PTP1B inhibitors can restore insulin sensitivity in diabetes; thus, PTP1B inhibitors validate this concept [118].

#### Takeda G-protein coupled receptor 5, GPBAR1

Takeda G protein-coupled receptor 5 (TGR5) also known as G protein-coupled bile acid receptor 1 (GPBAR1), is a membrane GPCR of bile acids and is expressed in L cells in the intestine, pancreatic beta cells, muscle tissue, brown adipose tissue, and macrophages. TGR5 activation elevates intracellular cAMP levels and activates the protein kinase A signalling pathway, with diverse metabolic consequences. Perhaps one of the more important functions of TGR5 is to enhance GLP-1 secretion from L cells in the intestine upon activation by bile acids or selective agonists. The increase in GLP-1 will facilitate greater insulin secretion from the pancreas (incretin effect) and suppress glucagon secretion, thereby helping maintain blood glucose levels. Moreover, TGR5 in brown adipose tissue and skeletal muscle could also elevate the metabolic rate and energy expenditure. This is related to increased local conversion of thyroid hormones and is likely beneficial in reducing obesity. Due to these two mechanisms together, TGR5 agonists are considered promising as a treatment for type 2 diabetes, especially in a patient with obesity. Certain TGR5 agonists have been shown in animal models to enhance glucose tolerance and insulin sensitivity while reducing hepatic steatosis, without significant systemic effects, particularly when their actions are directed primarily to the intestine [119].

The problem with targeting TGR5 is avoiding the gallbladder (as it may lead to cholecystitis) and cardiac stimulation. Thus, attempts to design TGR5 agonist molecules have been limited to compounds targeting the intestine. With the TGR5 (GPBAR1) structure known through homology modelling or recent crystallographic studies, docking and virtual screening approaches can be used to identify agonists. For example, natural compound libraries were screened for novel TGR5 agonist candidates using in silico methods [120]. All in all, TGR5 offers a remarkable focus that integrates the entire digestive system and glucose regulation, and, with the aid of computer simulations, the design and development of selective TGR5 agonists are expedited.

#### Insulin receptor and insulin receptor substrate-1

Insulin receptor (IR) is a receptor on the monocyte membrane, a heterotetramer whose tyrosine kinase activity is also activated by insulin. Insulin receptor substrate-1 (IRS-1) is a major cytoplasmic adaptor protein that is tyrosine-phosphorylated by IR when the insulin signal is active. They both form two significant features in the classical insulin signalling pathway. Insulin binding to IR triggers autophosphorylation of the receptor's tyrosine kinase domain, which subsequently recruits IRS-1, which is then phosphorylated. IRS-1 phosphoreguleted in this manner activates PI3K, which subsequently results in the phosphorylation of Akt/PKB and, in response, multiple downstream effects such as translocation of GLUT4 to the plasma membrane (enhancing glucose uptake) and modulation of metabolic enzymes (e.g. glycogen synthase). In this way, IR/IRS-1 acts as a bridge linking the insulin signal to cellular changes that regulate glucose. IR and IRS-1 functions are commonly altered under insulin-resistant states - for instance, an increase in IRS-1 levels or its functional tyrosine phosphorylation fails to occur and is instead serine phosphorylated (rather than tyrosine), leading to passive signalling blockade [114].

The positive modulation of IR/IRS-1 appears to be a rational approach to overcoming insulin resistance, as increasing its activity or concentration would enhance insulin signalling, increase glucose uptake in muscle and adipose tissues, and decrease hepatic glucose production. Insulin therapy, by contrast, provides exogenous insulin that activates IR and restores its “on” function. Activation of IR and some of its pathways has prompted screening for small, targeted molecules. There is also interest in the discovery of insulin mimetics that could activate IR via oral administration rather than injection. Several candidate compounds have been identified through cell-based and in silico docking assays targeting the IR kinase domain [121].

For instance, one investigation showed that a small molecule from microbial metabolism could bind and activate the β-subunit domain of IR, sullying IRS-1 phosphorylation mimicry of insulin action. While there has not been an effective “insulin pill” to date, these preliminary studies (aided by computational modelling and high-throughput screening) support the concept that, at some point, small molecules may be able to directly interact with the insulin receptor [121]. Another possibility is to enhance IR/IRS-1 signalling indirectly, for example, by inhibiting negative feedback controllers like PTP1B (as described in the PTP1B section above), which, as discussed before, has also been shown tentatively to bolster insulin signalling.

#### 11β-hydroxysteroid dehydrogenase type 1

11β-hydroxysteroid dehydrogenase type 1 (11β-HSD1) is an enzyme found within the endoplasmic reticulum of glucocorticoid target cells, such as hepatocytes, adipocytes, and muscle cells. It is an NADPH-dependent enzyme whose function is at the tissue level, where it converts cortisone (an inactive glucocorticoid) to active cortisol or, in animals, corticosterone from 11-dehydrocorticosterone. 11β-HSD1 therefore modulates the production of secreted cortisol in metabolic tissues, with less impact on circulating systemic cortisol levels. Cortisol enhances hepatic gluconeogenesis, increases fatty acid mobilization, and decreases insulin sensitivity in peripheral tissues. In visceral obesity and type 2 diabetes, 11β-HSD1 activity tends to be upregulated, thereby promoting overproduction of cortisol, which exacerbates insulin resistance and metabolic syndrome. This is corroborated by the finding that transgenic mice with overexpression of 11β-HSD1 in adipose tissue develop visceral obesity, insulin resistance, and glucose intolerance, mirroring the human metabolic syndrome. On the other hand, 11β-HSD1 knockout mice, which lack the enzyme that converts cortisone to cortisol at the tissue level, exhibit increased insulin sensitivity and are resistant to diet-induced obesity. These observations validated the pathophysiological role of 11β-HSD1 in diabetes and its associated metabolic disorders. Therefore, 11β-HSD1 inhibitors are proposed as possible treatments for T2DM and obesity by reducing local cortisol levels and improving insulin sensitivity without triggering systemic adrenal insufficiency [122].

Pharmaceutical companies have developed candidate drugs for 11β-HSD1 inhibition, including compounds from the carboxamide, triazole, and piperidinyl classes; however, some of these have failed in advanced clinical trials due to issues with efficacy and safety. Understanding the structure-activity relationships of enzyme inhibitors has been a focus of academic research. Important chemical features of potent 11β-HSD1 inhibitors have been mapped through combinatorial molecular docking and 3D-QSAR studies *in silico*. To illustrate, Murumkar *et al.* [123]. assessed the activity of 139 triazole and tetrazole derivatives as 11β-HSD1 inhibitors using active site docking of the enzyme and CoMFA/CoMSIA 3D-QSAR analysis. The outcome demonstrated a noteworthy structure-activity correlation, whereby specific substituents on the triazole skeleton increase the inhibition affinity. This knowledge aids in the rational design of novel inhibitors. In general, inhibition of 11β-HSD1 is hypothesized to reduce hepatic glucose production and increase hepatic insulin sensitivity. For this reason, there is ongoing interest in the development of novel antidiabetic drugs [123].

### Validation and limitations of in silico approaches

As with other disciplines in biomedical research, in silico methods are the first step in the discovery of new antidiabetic drugs. However, conclusions drawn from virtual information should be validated in physical laboratories and verified to ensure reliable outcomes. Within these methods, several gaps remain. Molecular docking, pharmacophore-based modelling, MD simulations, ADMET predictions and QSAR are perhaps the five most common methods used, each with its own form of validation and limitations. All these methods have been used in the study discussed below.

#### Molecular docking

Molecular docking aims to predict the binding interactions of a ligand, such as a drug candidate, with the active site of a target protein. Validation of docking results is typically performed by validating predictions and comparing them with experimental data. One of the more common approaches is to re-dock the native ligand to a protein whose binding sites are mapped from X-ray crystallography. The RMSD is calculated, and if the docked pose is close to the experimental pose (low RMSD), the method is validated. Furthermore, some test whether the docking affinity score and *the compound's in vitro activity* correlate, despite the known low correlation due to scoring limitations [124,125]. Validation also includes enrichment tests with benchmark compounds, in which active ligands are expected to rank higher than inactive or decoy counterparts. Ultimately, the ideal protocol for docking validation is to test predictions empirically by calculating correlations. As an example, prior to virtual screening, the accuracy of docking was assessed by redocking co-crystallized ligands, yielding an average binding energy error of approximately 8.368 kJ mol^-1^. Thus, reliance on docking energies alone for candidate selections would be inappropriate [124].

Technically, molecular docking has several fundamental limitations, including [124,125] :

1. Sampling of ligand and protein conformations is often limited; generally, proteins are assumed to be rigid (not fully flexible), so only a portion of the conformational space is explored.
2. The scoring function used is empirical or approximate, so that the predicted affinity value often does not match the actual affinity.
3. Docking depends on the availability of the target structure; if the crystal structure of the target protein is unknown, a homology model must be used, which can be less accurate.
4. Determination of protonation, handling of water molecules, and ion/co-factor interactions are often simplified, potentially leading to erroneous pose predictions.

Because of these issues, docking is prone to generating both false positives (inactive compounds with high scores) and false negatives. Some of these issues can be alleviated by using flexible docking, rescoring, or integration with MD simulations [125].

Regardless, molecular docking has proven usefulness in the discovery of antidiabetic drugs. For instance, virtual screening targeting the aldose reductase enzyme (linked to diabetes complications) employed docking-based methods to identify novel inhibitors among 53 screened compounds (~22 % success rate), six of which possessed unique chemical structures [124]. Although the subset was small, the notion that only a few compounds can contribute to the discovery of new chemotypes underscores the significance of the finding. In another case, docking studies of the PPAR-γ receptor showed that certain compounds had significantly higher binding scores than the native ligand, such as 5c and 5d, which were later shown to lower glucose levels in diabetic mice to levels comparable to those achieved with rosiglitazone [18]. The same study also utilized MD simulations to verify the stability of the ligand-PPAR-γ complex, which strengthened the confidence of docking results. These successful examples outline the applicability of docking as a primary screening tool, despite the need for subsequent experiments, in the development of new antidiabetic agents.

#### Pharmacophore modelling

Pharmacophore modelling (PhM) represents the interaction of ligands and targets as a three-dimensional model that includes features such as hydrogen-bond donor/acceptor, aromatic ring, and hydrophobic regions. Pharmacophore models can be built from the target’s structure (structure-based pharmacophore) or from a set of active ligands (ligand-based pharmacophore). Before using pharmacophore models for virtual screening, they must first be validated. Validation involves confirming the model’s ability to identify active molecules while rejecting inactive ones. In most cases, data are split into two groups: one comprising active (and sometimes inactive) ligands used to train the model, and another used to test the assumptions. Metrics such as hit rate, enrichment factor (EF), and Goodness of Hit (GH) serve as validation criteria. To illustrate, Chakravarthy derived a pharmacophore model for an Akt2 inhibitor and reported that it recognized all active ligands in the test set. However, it is equally important to ensure that the model does not identify inactive ligands. This is why researchers use decoy sets, which are largely random but contain a small number of hidden active compounds, for further verification. A good model is characterized by the ability to achieve significantly high concentrations of active compounds at the top-ranking positions when scanning this set [126].

In the study, a model achieved an active-compound retrieval rate of 16 out of 20, with a GH score of 0.72, on a pool of approximately 2000 decoys. This model's validity indicates that the pharmacophore is sufficiently sensitive and selective before being applied to a larger dataset. Moreover, self-validation using cross-validation or bootstrapping is performed on the active subset for ligand-based pharmacophore models to ensure the model isn’t biased toward the training set [126].

The complications of pharmacophore methods are often related to how a model simplifies the interactions of ligands and proteins. In structure-based pharmacophore methods, the model is often derived from a single protein structure, such as a crystal structure with a single bound ligand. Such an approach only reveals a snapshot of one binding mode, but in reality, proteins and ligands are dynamic: the active site undergoes shapeshifting (conformational flexibility), and multiple ligands can bind in various ways. Static models can omit crucial features of other ligands, resulting in false negatives (i.e., some active compounds are missed). Attempts to address this limitation include merging features from different protein-ligand complexes, when available, and applying dynamic pharmacophore models derived from MD trajectories. These dynamic pharmacophore approaches have been shown to improve virtual screening success compared with single rigid models, although they are more complex and require more computational power due to additional analyses, including model clustering [57].

In the context of antidiabetic drugs, pharmacophore methods have aided the development of new drug candidates. In 2019, Chang *et al.* [127] constructed a ligand-based pharmacophore model for SGLT2, a salt transporter used in the treatment of type 2 diabetes (T2D). After thorough validation, the model was employed for selective subset screening of a vast compound library, culminating in two identified hit compounds: one N-glycoside and a non-glycoside. Strikingly, both compounds demonstrated robust *in silico* binding affinity to SGLT2 and exhibited better pharmacokinetic and toxicity profiles than canagliflozin, an SGLT2 inhibitor. These results illustrate the utility of pharmacophore-driven multi-step *in silico* docking followed by ADMET analyses, offering promising new lead candidates for further pharmacological validation [127]. In other work, an integrated pharmacophore-Docking-QSAR model for the enzyme Dipeptidyl peptidase IV predicted, with high accuracy, unprecedentedly low-micromolar potent DPP-IV inhibitors. One such unanticipated result was gemifloxacin, a fluoroquinolone antibiotic, which exhibited an IC50 of 1.12 μM towards DPP-IV.

The finding that gemifloxacin was a DPP-IV inhibitor (and also an inhibitor of GSK-3β) emerged from an in silico screening study and demonstrates the potential of pharmacophore/QSAR models to reveal hidden drug repurposing opportunities [128]. Such outcomes provide evidence supporting the pharmacophore approach, provided it is well-designed and rigorously analysed, and simultaneously underscore the necessity of integrating other techniques to compensate for their individual shortcomings.

#### Molecular dynamics simulations

Molecular dynamics (MD) simulations now track the motion of atoms in biological systems, such as protein-ligand complexes, over time; therefore, they add a dynamic component that is missing in static models. In verification processes, MD simulations are often employed as a final step to confirm the temporal stability of a docked protein-ligand complex. The MD simulations run for several tens to hundreds of nanoseconds, and the principle is simple: if the proper pose is stable, the ligand will be retained within the active pocket, with key contacts maintained. On the other hand, if the bound pose is erroneous (weak affinity or incorrect orientation), the ligand tends to drift out or fully dissociate from the active site during MD. This was elegantly illustrated by Sakano *et al*. [129]: in a flexible system with numerous ligands, post-docking MD simulations allowed incorrectly placed ligands to escape from their binding sites, whereas rigid systems with conformationally homologous ligands as templates tend to maintain stable docking poses. In essence, this suggests that MD simulations may serve as validation filters, in which “comfortable” poses are attained with minimal conformational change, whereas incorrect poses undergo substantial movements, signalling unstable intermediate structures.

In addition to performing stability checks, MD also enables more accurate binding energy calculations using methods such as MM-GBSA or free-energy calculations, whose results can be compared with experimental data to validate predicted affinity sequences [129].

Boundaries of MD simulations are constrained by temporal scope, force-field accuracy, and computational burden. Within too short a timeframe, only brief, localized conformational shifts, such as side-chain twists and micro vibrations, are captured, whereas more critical slow dynamics are completely missed. For instance, slow rearrangements of protein loops or allosteric shifts likely occur on timescales of microseconds to milliseconds, which are far beyond those routinely sampled in nanosecond simulations. Because of this restriction, poses that appear “frozen” and energetically minimal in 50 ns MD simulations likely do not reflect long-lived biological relevance; they could snap out of that pose when removed from the time-constrained simulation. Despite advances in hardware (GPUs, parallel computing) that increase the scale of MD simulations [116], the trade-off in computational cost remains significant when numerous candidates are simulated. Additionally, widely used classical force fields, such as AMBER and CHARMM, employ assumptions, including fixed static charges, that neglect the explicit modelling of electronic polarization. This diminishes the accuracy of certain interactions, such as ionic interactions, in which the zealous shift in the environment due to ionization is only partially accommodated. While polarized force fields are available, their implementation remains expensive and complex.

As with enzymes and chemical reactions, classical forces do not permit bond formation; therefore, metabolic or covalent processes cannot be simulated without QM/MM techniques [130]. In addition, MD simulations require specification of the starting protonation state and pose; all subsequent results are virtually guaranteed to diverge from biologically relevant routes if the initial guesses, such as different active ligand tautomers, are incorrect. Yet another restriction is the understanding of “big data” MD data: simulations not only output MD trajectories but also produce thousands of conformations that must be filtered using clustering or PCA to identify coherent patterns.

Regardless, MD has proven highly useful in antidiabetic drug research, particularly for validation and optimization. For instance, a docking study of a flavonol compound to the SUR1 receptor (pancreatic K-ATP channel complex) reported that 100 ns MD simulations showed that ligand-SUR1 complexes form, capturing verification of critical interactions that “warming up,” which confirmed crucial interactions and validated the hypothesized mechanisms underlying the compound’s activity [131]. In another example mentioned and described earlier in this discussion, 150 ns MD simulation of a PPAR-γ complex with a novel ligand demonstrated convergence of similar behaviour of RMSD and interaction distances to those of the native ligand complex, confirming stable binding of the novel ligand. The simulation results agreed with experiments performed on a model of adult mice using the compound, which successfully reduced blood sugar levels without marked side effects [18]. MD simulations may pose significant challenges but have become an essential part of today's *in silico* validation and prediction, helping determine which protein-ligand complexes should undergo further detailed synthesis and biological screening.

#### ADMET

ADMET prediction model validation is performed by benchmarking the experimental data (*e.g.* gut permeability, metabolic stability, and animal toxicity) for known compounds against the predicted results. Many ADMET models are developed using QSAR or machine learning and rely on large datasets, so they often employ internal cross-validation and external test-set evaluation. A toxicity prediction model, for instance, should be able to accurately classify the vast majority of both toxic and non-toxic compounds in the test set (*e.g.* ROC AUC and balanced sensitivity/specificity). Rigor validation remains tied to the OECD QSAR protocols, under which the model must specify a clear validity domain, along with external stability and predictivity. ADMET differs from other disciplines due to the higher number of interdependent parameters that are intricately unique - there is not one experimental “number” to verify (for instance, human oral bioavailability, plasma half-life, hERG binding potential, Ames mutagenicity risk, *etc.*). Thus, validation is typically conducted using a series of submodels (log P, Caco-2 permeability, and liver toxicity) based on historical data. These models are also empirically evaluated on public datasets despite their varying quality.

An industry study by Bayer emphasized that the performance of ADMET models is heavily dependent on their training data. During the early 2000s, data for each endpoint were only a few hundred compounds; however, now these datasets have been expanded to thousands of homogenous data points for each endpoint to strengthen model robustness. The reliability of ADMET machine learning models in predicting new compounds increases significantly when rich, curated internal data are available. This strong internal data helps improve model predictions, as demonstrated by the p*K*_a_ and site of metabolism prediction accuracy after the incorporation of thousands of high-quality data [132]. While *in silico* validation is critical, experimental validation always remains paramount. Assume a compound's *in* silico prediction shows no toxicity and favourable bioavailability; this must be verified with in vitro assays *(e.g.* hepatotoxicity and membrane permeability assays) as well as animal studies before advancing to subsequent development stages. Therefore, this indicates that ADMET models are primarily used to replace tests rather than to eliminate them [133].

Despite limitations, integrating ADMET predictions into antidiabetic therapeutics research has proven productive. Numerous projects in drug discovery employ in silico ADMET profiling during the hit-to-lead stage to refine candidate optimization. Consider the previously discussed SGLT2 inhibitor discovery, in which two pharmacophore screening hits suggested not only strong target affinity but also predicted superior pharmacokinetic parameters: both were predicted to have high oral bioavailability and were metabolically stable, with no toxicity alarms (including low hERG potency), compared with the standard canagliflozin [127]. These predictions helped justify advancing the candidates to preclinical trials with confidence and minimal risk of adverse effects. In another study, a synthesized derivative of the sulphonamide, designed as a prospective antidiabetic drug, underwent *in silico* ADMET evaluation and its predicted profile indicated that it was membrane permeable (not a P-gp substrate), crossed the blood-brain barrier moderately, and was non-mutagenic in the Ames test [134]. This type of data helps researchers direct biological assays toward the most promising compounds.

Nonetheless, there are instances in which ADMET predictions are overlooked. For instance, a certain antidiabetic drug may pass all *in silico* evaluations, but later (in clinical trials), it may cause rare side effects like pancreatitis, or any other side effect whose mechanism of causation is not represented in the model. This serves as a reminder that ADMET models are simplifications and do not capture the full picture of human physiology. In any case, the use of *in silico* ADMET models has been shown to reduce the rate of late failure by filtering out “problematic” compounds early, provided the operator understands the model's limitations and continues to perform laboratory validation.

#### Quantitative structure-activity relationship

The limitations of a QSAR study lie in the data, methodology, and interpretation of results. Biological data input is often the most critical step, as experimental errors in biological data collection can obscure true structure-activity relationships. If a compound is assigned an incorrect activity value, it can severely jeopardize the model's predictive performance. Moreover, traditional QSAR approaches are best suited to congeneric compound series that share a common mechanism of action; once the dataset comprises compounds with multiple divergent modes of action, a single linear fit is insufficient. This is illustrated by the DPP-IV case described above. The analysis identified two orthogonal pharmacophores that were weakly interdependent among a set of DPP-IV inhibitors, suggesting they occupy two distinct binding modes. One model would not adequately describe such multi-modal systems; therefore, those authors used both pharmacophores in a multiparameter QSAR model. Additionally, boundary overlaps are important in other domains: QSAR models are constrained to specific chemical spaces. There is a certain limit on flexibility beyond which antagonistic scaffolds cannot be safely predicted.

For this reason, QSAR requires extreme caution when devising new analogues, as significant structural deviations may render the model “blind”. Overfitting can exceed a model's target accuracy when the number of descriptors increases while the data remain the same; this can be mitigated by enforcing *N*/*Q* >= 5, where *N* denotes the number of compounds and *Q* the number of free descriptors. While contemporary techniques such as regularization or ensemble methods reduce overfitting, none completely eradicate it. Moreover, the predictive capability of QSAR differs. Simple linear models like Hansch analyses are straightforward and each parameter is a simple representation of the log *P* and Hammett sigma contribution. Using random forests, support vector machines, and neural networks (all within the broader category of machine learning), the model provides predictive performance but does not explain which chemical details drive the predictions. While these models can be partially explained by citation-based importance or Shapley value techniques, which assign contribution values to effects, their lack of transparency relative to structural methods, such as docking, limits their utility. Lastly, pharmacokinetics and other relevant factors are often overlooked in QSAR modelling.

In addition to an ADMET-focused QSAR, an ADMET-specific model, combined with the preliminary QSAR, would be needed to address compounds that fail to progress to drug development due to large discrepancies in predicted potency.

Nonetheless, the development of antidiabetic candidates and diabetes treatment has greatly benefited from the use of QSAR. The construction of some historical megulitide derivatives is a marked example resulting from the Hansch analysis of benzoic acids, which enhanced the efficacy of novel sulfonylurea receptor agonists. Over the past ten years, active unexpected molecules have been identified through a combination of virtual screening and QSAR methods. As noted before, the pharmacophore model incorporating DPP-IV 1 showcased significant predictive capabilities by identifying gemifloxacin as a highly potential DPP-IV inhibitor [128].

Antidiabetic drug design still utilizes QSAR techniques as a supplement to methods based on the drug's structure. Experimental confirmation remains the undisputed gold standard for validating QSAR-generated models; however, with a robust model in place, QSAR can expedite lead discovery by pre-emptively filtering thousands of candidates to the most viable ones. The integration of QSAR with docking and other methods, such as pharmacophore models and ADMET analyses, into a single computational design pipeline forms a complete system: QSAR estimates the likely highest-value compounds, docking with MD confirms binding and selectivity hypotheses, and all ADMET models evaluate drug-likeness. Using multiple methods increases the likelihood of success in real-world testing [125]. Scientists will be encouraged to improve in silico methodologies and refine experimental workflows for safe, effective antidiabetic drugs by comprehensively assessing each technique’s validation potential and the limitations of each method.

## *In silico* exploration of antidiabetic compounds: a source-based review

### Compounds from plants (ethnopharmacology)

Ethnopharmacology relies on traditional knowledge to investigate bioactive compounds of antidiabetic plants. In Indonesia, for example, more than 130 species of traditional plants are used to treat diabetes [135]. Tropical plants are also known to contain high levels of secondary metabolites (e.g., flavonoids, alkaloids, phenolics), which lower blood glucose levels by inhibiting α-glucosidase or DPP-IV, or by activating PPARγ. For example, a new arylbenzofuran compound from *Morus mesozygia* (African mulberry) is traditionally used for diabetes and was reported to have high binding affinity for α-glucosidase (Δ*G* = -39.75 and -36.40 kJ mol^-1^) [123], supporting the ethnopharmacological claim. Therefore, ethnopharmaceutical plants have proven to be an important source of new antidiabetic compounds.

Various computational techniques (*in silico)* are applied to elucidate these plant compounds. Molecular docking foresaw the pose and affinity of active molecules in the target protein's active region [136,137], while molecular dynamics (MD) simulations monitor ligand-receptor complex stability over a given period (RMSD/RMSF analysis) [138]. To evaluate the pharmacokinetic properties and toxicity of the claimed phytochemicals, natural compounds were screened using ADMET models [139]. With pharmacophore modelling, one focuses only on the critical molecular traits likely to interact with the target, in contrast to QSAR, which associates the geometric or electronic structure of a molecule with its reactivity or biological activity [82].

Normally, the *in silico* steps begin by selecting specific medicinal plants from ethnopharmacology literature and determining their key phytochemicals, screening for drug-likeness and ADMET parameters, predicting possible interactors, performing docking on diabetes targets like DPP-IV, α-glucosidase, PPARγ, SGLT2 and so on, and validating using MD simulations including binding free energy calculations with MM-PBSA [82]. This sophisticated structure facilitates efficient screening while narrowing the pool of prospective candidate compounds. Several recent studies demonstrate the application of these processes [139-141].

Over the past five years, numerous studies on plant-derived compounds have been conducted, as presented in Table 1. The graph shows a surge in interest in the extraction of natural molecules as potential antidiabetic agents, evaluated using in silico methods. Most of the identified compounds belong to the classes of flavonoids, alkaloids, and terpenoids; each of these groups is generally strongly associated with key enzymes such as α-glucosidase, DPP-IV, and PPARγ. The investigations employed molecular docking techniques, ADMET predictions, and several in vitro or in vivo validations. The summary in Table 4 highlights the potential of phytochemicals as antidiabetic agents and the utility of computational approaches in the early stages of drug discovery.

**Table 4.** *In silico* research of antidiabetic compounds from natural products. This research targets one or more target proteins related to the mechanism of diabetes, either inhibition or induction

Compound	Protein target	Methods	Result	Ref.
Gluggusterone E (from *C. mukul*)	DPP-IV	Molecular Docking	Higher binding affinity than vildagliptin (Δ*G* more negative)	[136]
*Ammannia aegyptiaca* (ethanolic extract of flowers), active compound: myricetin 3-O-(6″-O- -galloyl)-β-glucopyranoside 7-O-β-glucopyranoside (MGGG)	α-amylase	Docking (CDOCKER); *in vivo* test (mouse); ADMET	MGGG compound exhibited a strong interaction with α-amylase (active site) having a binding energy of Δ*G* = -37.61 kJ mol^-1^ which is much better than that of acarbose (-21.09 kJ mol^-1^). The *in vitro* and *in vivo* studies verified the antidiabetic activity: AEEE extract diminished blood glucose levels and serum α-amylase activity in diabetic rats. Docking analysis clarified the persistent hydrogen bond interaction at the active residue. MGGG and AEEE extract formulate-also supports the mechanism of action for α-amylase inhibition demonstrating potential antidiabetic efficacy	[137]
Berberine	FOXO1	Molecular docking and MD	The docking results revealed that berberine had a binding affinity value of -25.56 kJ mol^-1^, stronger than metformin's -20.17 kJ mol^-1^ and close to FOXO1 inhibitor AS1842856 which has an affinity of -28.66 kJ mol^-1^. For berberine, the MMGBSA binding free energy is calculated at- 107.78 kJ mol^-1^, showing significantly greater binding efficiency than metformin’s -18.37 kJ mol^-1^thereby suggesting a more thermodynamically stable complex for berberine. In the molecular dynamics simulation over 500 nanoseconds, the RMSD and RMSF values for key residues Arg7, Ala10, Lys22, Ser44, Val45, Pro46, and Tyr47 showed minimal fluctuation while remaining consistent with stable hydrogen bonds which reflects that berberine maintains high structural stability of the FOXO1-berberine complex enabling quantitative and stable inhibition of transcription function and gluconeogenesis activity	[41]
14-Deoxo-14-O-acetylorthosiphol Y (from *O. stamineus*)	SGLT2 & SGLT1	Molecular docking, MD and ADMET	Δ*G* = -47.70 kJ mol^-1^ (SGLT2); stable complex (RMSD ≈0.48 nm); favourable ADMET profile	[139]
8-C-Glucopyranosyleriodictylol (from *P. macrocarpa*)	α-glucosidase	Docking and MD	Highest affinity among 14 test ligands (better than standard control)	[142]
Moracin P/M (from *M. mesozygia*)	α-glucosidase	Molecular docking	Affinity -39.75 and -36.40 kJ mol^-1^ (stable at active site)	[143]
Isolate of ethyl acetate fraction of *Ficus lutea* leaves: epiafzelechin, epicatechin, lupeol, stigmasterol, α-amyrin acetate	α-amylase, α-glucosidase, glycogen phosphorylase, PPARγ, DPP-IV, glucokinase, PTP1B, GLUT1	Molecular docking (AutoDock Vina), ADMET profile	All five isolated compounds demonstrated high binding potential to the diabetes targets. For GP (1NOI) and α-amylase (1OSE), the binding energy values were lower than the benchmark control also positive at ≈ -33.89 kJ mol^-1^. Stigmasterol exhibited the most negative binding energy and was the most versatile ligand, binding to five out of eight targets, while lupeol bound to three prime targets. ADMET: epiafzelechin and epicatechin did not break Lipinski's rule (good drug profile) whereas stigmasterol, lupeol and α-amyrin acetate infringed due to high log*P*	[62]
Bioactive compounds of *Cissampelos pareira* (synthetic ligands)	DPP-IV, PPARγ, α-amylase, α-glucosidase, GK, PTP1B, GSK3β	Molecular docking (AutoDock Vina)	Among the screened derivative compounds, some exhibited higher binding affinity than quercetin (control). For instance, ligand 15 had affinities of -36.82 kJ mol^-1^ to DPP-IV (compared to quercetin’s -9.1), -10.1 to PPARγ; both ligands 23 and 31 showed -43.10 kJ mol^-1^ to PPARγ and ligand 26 to PTP1B had -38.91 kJ mol^-1^ ligand 15 also showed strong binding to α-amylase and DPP-IV. These compounds are suggested as antidiabetic proposals because of their low binding energy to several important targets	[144]
Polyholistic mixture (*Vernonia amygdalina* leaves, *Allium sativum*, *Ocimum gratissimum*), GC MS compounds: stigmasterol, γ-sitosterol, tocotrienol (vitamin E)	α-glucosidase	Consensus docking (SAMSON, PyRx, iGEMDOCK); ADMET	After performing docking analysis and GC MS, it was found that stigmasterol, γ-sitosterol, and tocotrienol have the most significant interactions with the active site of α-glucosidase outpacing acarbose at 4th position. These interactions are characterized by hydrogen bonding and van der Waals interactions. In terms of ADMET prediction, all three compounds were shown to comply with the criteria ‘ drug-likeness’ (do not violate >1 criteron), while also being non-hepatotoxic, non-carcinogenic, non-AMeS, and hERG negative which makes them biologically safer than acarbose. This demonstrates that all three compounds can be considered as potential natural α-glucosidase inhibitors	[145]
*Dalbergia sissoo* (bark extract) - candidate compounds: soyasapogenol B, korydin	α-amylase, α-glucosidase, DPP-IV	Molecular docking (AutoDock), ADMET and MD (permeabilitas membrane)	Both compounds soyasapogenol B and corydin exhibited remarkable binding with energies surpassing expectation: soyasapogenol B bound to α-amylase with Δ*G* ≈ -54.40 kJ mol^-1^, which is more negative than acarbose’s value of -32.22 kJ mol^-1^. Molecular dynamics simulation over 100 ns showed a stable complex for α-amylase with soyasapogenol B and corydin, with RMSD <0.3 nm for the backbone. Membrane permeability test (PerMM) estimation predicted better penetrative capability for the soyasapogenols compared to acarbose and sitagliptin. Prediction of ADMET data: these compounds obey Lipinski’s rule suggesting favourable bioavailability. All in all, the two compounds together suggest they should be further investigated as anti-diabetics	[140]
Palmatine (*Fibraurea tinctoria*)	DPP-IV	Molecular docking	Palmatine demonstrates strong binding affinities to two important diabetes management enzymes, alpha-glucosidase (with a binding affinity of -25.52 kJ mol^-1^) and DPP-IV at -29.71kJ mol^-1^. This indicates stable interaction and inhibition potential. The more negative affinity value for DPP-IV confirms stronger binding when compared to alpha-glucosidase. Collectively, these findings highlight palmatine's capabilities as a natural anti-diabetic compound which can inhibit the activity of key enzymes involved in blood glucose concentration regulation	[146]
*Morus mesozygia* (root) - active compound: Moracin P (prenylarilbenzofuran)	α-glucosidase	Molecular docking (AutoDock 4/Vina)	Arylbenzofuran moracin P (compound 2) exhibited the highest binding affinity to α-glucosidase with a value of Δ*G* =- 39.75 kJ mol^-1^, significantly lower (stronger) than that of acarbose (-34.73 kJ mol^-1^). Other compounds (1 and 3) were also notable in their effectiveness (-36.40 and -35.56 kJ mol^-1^ respectively). All compounds bind through hydrogen bonds and π bond interactions with the relevant active site residues. This docking analysis confirms the proposed inhibitory action of α-glucosidase and elucidates the *in vitro* effect on urinary glucose excretion	[143]

### Synthetic and analogues of standard compounds

The development of new synthetic compounds and analogues of standard antidiabetic drugs is intended to enhance their efficacy. For instance, the use of α-glucosidase inhibitors such as acarbose, miglitol, and voglibose decreases postprandial glucose levels, but is associated with gastrointestinal bloating and diarrhea nature.com. There is also limited efficacy and stability with sulfonylureas (glibenclamide) and biguanides (metformin). Therefore, to increase target affinity and reduce toxicity, structural modifications are made to standard compounds such as metformin analogues, glibenclamide derivatives, and thiazolidinedione acid group conjugates. This helps provide deeper analysis of novel chemical scaffolds and extends drug patent periods [147]. During the initial phases of drug development, an *in silico* approach is crucial, as it can efficiently screen thousands of analogues before laboratory synthesis.

Gul *et al*. [148] synthesized new analogs of α-glucosidase inhibitors, known as “bis-Schiff derivatives”, and furthered the studies with *in silico* approaches to design their antidiabetic derivatives. Two of these compounds (8 and 9) had approximately nanomolar α-glucosidase IC_50_ values (better than the clinically used drug acarbose, which has an IC_50_ of 0.27 μM). Docking studies showed that compound 10 had a binding energy of -45.61 kJ mol^-1^ with α-glucosidase, confirming that a strong ligand-target interaction was present. They also reported another derivative with an IC_50_ of ~12.8 μM, approximately 7,000-fold more potent than acarbose. This study employed molecular docking to characterize the binding poses and interactions of the compounds with the enzyme.

As another example, Zahra *et al*. [149] synthesized thioscarbazone derivatives from coumarin (compounds 3a to 3m). These compounds showed much stronger inhibition of α-glucosidase than acarbose (IC_50_ = 2.33 to 22.11 μM, while acarbose’s IC_50_ was 873 μM). The most potent member from this group was compound 3c (IC_50_ = 2.33 μM). Molecular docking studies showed that compound 3c had a score of ≈ -26.53 kJ mol^-1^, the best in the group and consistent with its in vitro activity. When all these results are combined, these new compounds can be proposed as antidiabetic drug candidates, with in silico studies suggesting they may be more effective at lower doses.

An overview of *in silico* investigations over the past five years is presented in Table 5, which discusses various synthetic and analog antidiabetic molecules.

**Table 5.** *In silico* research of antidiabetic compounds from synthetic and analog compounds. *in silico* exploration of synthetic antidiabetic compounds often begins with established pharmacophores or bioactive scaffolds. Researchers modify the templates *in silico* to refine binding affinity, boost specificity, and improve the predicted pharmacokinetic profile. The table lists molecular targets alongside docking scores and ADMET projections, allowing a side-by-side comparison of predicted structure-activity relationships. Observed trends suggest several candidates may outperform existing drugs once bench studies are completed

Compound	Protein target	Structure	Methods	Results	Ref.
Thiobarbiturate-based bis-Schiff base (compound 10)	α-glucosidase	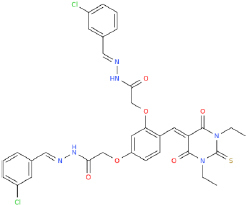	Molecular docking (Vina), DFT calculation	IC_50_=0,10 μM; Δ*G* bind = -45.61 kJ mol^-1^	[148]
Hydrazide-hydrazone derivative 3,4-dihydroxyphenylacetate (compound 5)	α-glucosidase	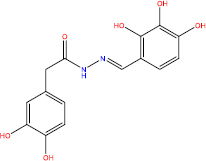	Molecular docking (Vina)	IC_50_=12.8 μM (acarbose=873 μM); docking shows hydroxyl interaction	[150]
3,6,7-triacetyl-ester-γ-mangostin	α-glucosidase	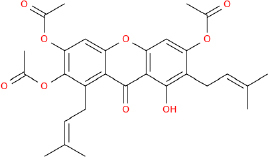	Molecular docking, and MD	Mangosteen and its derivatives developed the strongest antidiabetic properties by having the greatest binding affinity with α-glucosidase enzyme. γ-mangostin’s binding value was better than acarbose, surpassing -28.87 at -33.47 kJ mol^-1^. Derivative 3,6,7-trimethyl-ester-γ-mangostin also showed good affinity of -30.54 kJ mol^-1^ and the lowest binding free energy from molecular dynamics simulation, -132.26 kJ mol^-1^, much more stable than acarbose which had -72.47 kJ mol^-1^. The combination of 3,6,7-trimethyl-ester-γ-mangostin with maltose also demonstrated a competitive free energy of -87.36 kJ mol^-1^ supporting noncompetitive inhibition with stable constraining feedback mechanisms without changing the system dynamics significantly. All these numbers confirm that mangostin and his derivatives are more effective compared to acarbose which makes them useful for treating diabetes	[138]
7-fluorochromone-thioscarbazone derivative (compound 3m)	α-glucosidase	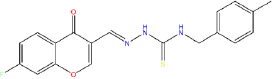	Molecular docking (Vina), MD, ADMET	IC_50_=6.40μM; docking Δ*G* ≈ -34.73 kJ mol^-1^	[147]
Coumarin-thioscarbazone derivative (compound 3i)	α-glucosidase	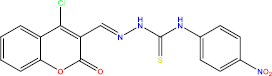	Molecular docking (MOE), MD	IC_50_=2.13±0.04 μM; docking Δ*G* ≈ -28.33 kJ mol^-1^	[149]
Benzimidazole derivative-Sebase (compound 8p, thiophene-2-yl substituent)	α-glucosidase	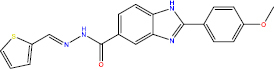	Molecular docking (glide), MD, ADMET, QSAR	IC_50_=70.6 μM (carbose 750 μM); complex RMSD ≈0.17 nm; satisfactory ADMET profile	[151]
2-(4 nitrophenoxy)isobutyric acid (compound 2)	PPARγ	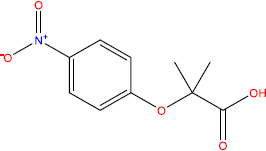	Docking, MD, ADMET	Docking revealed Δ*G* to be approximately -32.64 kJ mol^-1^, while MD simulation over 50 ns confirmed stable complex formation with Δ*G* around -30.54 kJ mol^-1^. The ligand establishes critical hydrogen bonds with His-323, Tyr-473, His-449, which suggests PPARγ agonism. ADMET analysis showed low toxicity risk with low hERG blockade and moderate CYP enzymatic clearance	[141]
Steroidal pyrimidine analog 9a (compound 9a)	SGLT2	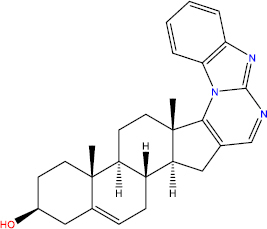	Molecular docking, MD (MM/GBSA)	Docking studies and MD simulations revealed the significant interaction at the glucose entry site of SGLT2. Averaged over more than 100 snapshots, the binding energy obtained from MM/GBSA was approximately -215.97 kJ mol^-1^ and the RMSD profile was less than 0.16 nm, suggesting a stable protein-ligand complex throughout the simulation. Ligand 9a obstructs the glucose channel and makes extensive interactions with Glu99, Asp454 (H-bonding more than 97 % of the time), and Phe453 (π-cation interactions 69 % of the time). The ADMET predictions of compound 9a suggest that it satisfies Lipinski’s rule of five, ASI >90 %, low exposure to the blood-brain barrier, and non-carcinogenic properties	[152]
Thiosemicarbazone *3c*	Aldose Reductase (ALR2)	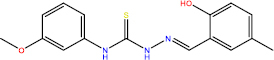	Molecular docking, MD, QSAR, ADME	According to glide docking results, Δ*G* ≈ -37 kJ mol^-1^ (for compound 3c with ALR2). This value corresponds to hydrogen bonds with Trp111, hydrophobic contact with Val47, Tyr48, Phe122 etc. MD simulations for 50ns (MD) showed stable binding for the ligand in the active site. In the case of disadvantageous docking poses, the active site entry might be energetically costless; however, averaged calculations lowered the Δ*G*_bind to ≈ -25 kJ/mol for the first cluster in MM/GBSA evaluation (MM/GBSA). Predictions from QSAR/QSPR models along with the ADMET analysis indicated favorable pharmacokinetic profiles: good solubility, ADME-friendly metrics, and no acute toxicity concerns	[153]
3-(Benzylsulfamoyl)-5-nitro-N-(1,3-thiazol-2-yl)benzamide (6h)	Glucokinase	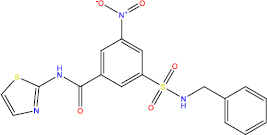	Molecular docking (glide)	Glide docking using the allosteric site of GK provided a Glide score of -11.11 along with a binding energy of -239.28 kJ mol^-1^ for compound 6h, which indicates a very high affinity. Ligand 6h parallels the position of co-ligand (3IMX) within the allosteric pocket, binding through H-bonds to Arg63 and π interactions with adjacent hydrophobic residues (Tyr214, Met210, Val455). The *in vitro* measurement of GK activation was in the range of 1.8 to 2.1× the control value.	[154]
DCCT13 (coumarin-chalcone hybrid)	Insulin receptor	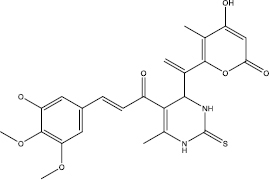	Molecular docking	*In silico* docking (PDB:1IR3) indicated that DCCT13 was the lowest scoring ligand in the analog series, thus, exhibiting the highest binding affinity to IR. The DCCT13 ligand exhibited strong interactions with the catalytic domain of IR. Further *in vivo* studies substantiated the antihyperglycemic effects of DCCT13 (reduction in blood glucose levels), thereby reinforcing the *in silico* findings of its predicted utility as an IR antagonist	[155]

Researchers typically begin with a well-established pharmacophore framework and then apply computational modifications to enhance binding affinity, target specificity, and pharmacokinetic properties. The α-glucosidase enzyme, the PPARγ receptor, and the SGLT2 transporter remain the primary therapeutic targets, each contributing directly to blood glucose control. Various approaches, such as molecular docking, molecular MD, QSAR and MM/GBSA calculations, were used to explore these interactions. In practice, several compounds, such as coumarin derivatives and thiobarbiturates, have IC_50_ values lower than those of acarbose, indicating promising inhibitory potential. Further validation using molecular dynamics simulations confirmed that the ligand-protein complex remained stable throughout the simulation. Finally, the satisfactory ADMET profiles suggest that the candidates listed in this table are worthy of experimental testing to expand the fleet of new-generation antidiabetic therapies.

### Compounds from database

Computational databases contain collections of chemical compounds that span a wide range, aiding the efficient discovery of antidiabetic drugs. For example, the ZINC database contains billions of purchasable compounds ready for virtual screening [36], while PubChem contains over 116 million unique chemical structures with approximately 297 million bioactivity data [156]. Curated databases such as ChEMBL contain bioactive compounds and associated literature data, enabling QSAR modelling and ligand-based screening. Furthermore, natural product databases such as NPASS, IMPPAT, TCMID, SuperNatural and AfroDb specifically compile phytochemicals of traditional medicines, as well as natural metabolites of herbal drugs [157,158]. Such compounds are important because many antidiabetic drugs have herbal origins; NPASS alone provides thousands of natural compounds tested for bioactivity, including antidiabetic activity [158], while IMPPAT (Indian Medicinal Plants, Phytochemistry and Therapeutics) offers information on thousands of phytochemicals from medicinal plants [157].

In a recent study by Ndarawit *et al.* [158], the authors screened a total of 30,926 compounds from the NPASS database, emphasizing drug-likeness, toxicity profiles, and performing docking on alpha amylase and alpha glycosidase enzymes. This work identified two molecules, such as NPC204580, which exhibited the lowest binding energy and served as dual inhibitors, with good complex stability, as shown in the 100 ns MD simulation. Zare *et al.* [159] conducted a virtual structure-based screening of millions of ZINC compounds for DPP4 targets, validating the best candidates through docking, MD, and ADMET analyses. Compound ZINC000003015356 was found to be a selective DPP-IV inhibitor showing lower binding free energy than the crystal ligands. Aldahish *et al.* [160] described the modelling of a diabetes-related GLUT4 receptor to which they pre-docked approximately 5000 ChEMBL/ZINC compounds, stating that ligand ZINC000216155214 retrieved the highest score, deeming it a potential inhibitor. As for Shahab *et al.* [161], they obtained roughly 100,000 compounds from SANCDB/ /AfroDb, along with their own collection. He then screened these against aldose reductase using SBVS (AutoDock4) coupled with MD; the two best compounds, ZINC35671852 and ZINC78774792, were identified based on their docking scores. Rashid *et al.* [162] evaluated 51 FDA-approved drugs from the ZINC database and conducted docking and MD studies to assess their activity against α-glycosidase, identifying Trabectedin (ZINC000150338708) as a potent inhibitor with an IC_50_ of approximately 1.26 μM.

Table 6 summarizes a series of *in silico* investigations conducted on antidiabetic compounds retrieved from prominent public repositories, including ZINC, NPASS, ChEMBL, AfroDB, TCMID, and IMPPAT. Virtual screening emphasizes the alignment of compounds' three-dimensional scaffolds with the catalytic or allosteric pockets of proteins central to the onset and progression of type 2 diabetes. In most cases, the entries are drawn from natural product libraries, semisynthetic analogs, or plant-derived metabolites, whose biochemical profiles have already been experimentally validated. The criteria for the first-round filtering included geometric and electronic features predictive of strong occupancy in the active sites of enzymes and transporters, such as α-glucosidase, α-amylase, DPP-IV, GLUT4, SGLT2, aldose reductase, PTP1B, and the MAPK1-PI3K cascade.

**Table 6.** *In silico* research of antidiabetic compounds from the database. The compounds usually choose potential molecules by first evaluating how well their three-dimensional scaffolds nest into the active sites of the central diabetic proteins. That preliminary fit is then tested through molecular docking runs alongside in-silico ADMET and basic pharmacokinetic screens

Compound	Protein target(s)	Method(s)	Result	Data sources	Ref.
NPC204580 (chrotacumine C)	α-amylase, α-glucosidase	Molecular docking, MD, ADMET	Identification of dual potent inhibitors with low free binding energy; stable complex (MD 100 ns)	NPASS	[158]
ZINC000003015356	DPP-IV	Moleculard, MD, MM/PBSA, ADMET, DFT	Selective DPP-IV inhibitor (lower binding energy than control ligand); stable complex	ZINC	[159]
ZINC000216155214	GLUT4	Molecular docking, ADMET	Highest docking score on GLUT4 target; potential inhibitor candidate	ZINC, ChEMBL	[160]
ZINC35671852	Aldose Reductase	SBVS, Molecular docking, MD (MM/PBSA)	Selected as top hit; docking score ~ -31.39 kJ mol^-1^; strong interaction at active residues	ZINC	[161]
Trabectedin (ZINC000150338708)	α-glucosidase	Molecular docking, MD	Potent α-glycosidase inhibitor (IC_50_ ≈ 1.26 μM); stable binding (Δ*G*_bind ≈ 309.61 kJ mol^-1^)	ZINC (FDA drugs)	[162]
CH0002 (ChEMBL ID)	DPP-IV	QSAR/AI screening, molecular docking	Identified as having high binding affinity for DPP-IV and low for DPP8/9, indicating selective potential as a DPP-IV inhibitor	ChEMBL	[163]
NPC204580 (NPASS)	α-amylase (3BAJ) dan α-glucosidase (2QMJ)	Molecular docking structure (SBVS), MD	Docking scores of -60.50 kJ mol^-1^ for α-amylase (3BAJ) and-35.23 kJ mol^-1^ for α-glucosidase (2QMJ), are more negative than acarbose; ligand RMSD values <0.2 nm, indicating stable complexes in MD	NPASS	[158]
NPC137813 (NPASS)	α-amylase (3BAJ) dan α-glucosidase (2QMJ)	Molecular docking (SBVS), MD	Docking scores of -52.63 kJ mol^-1^ for α-amylase (3BAJ) and 36.65 kJ mol^-1^ for α-glucosidase (2QMJ), compared to acarbose -12.99 and -8.22; the ligand-protein complex is stable (RMSD <0.2 nm) in MD simulations	NPASS	[158]
(+)-pipoxide (AfroDB)	Aldose reductase	Molecular docking SBVS, ADMET, MD, MM/PBSA	High binding affinity; docking scores ranging from -51.46 to -44.77 kJ mol^-1^ (better than standard inhibitors); strong interactions (hydrogen and hydrophobic) with key AR residues; good ADMET profile (low toxicity); high stability in MD and MM-PBSA, confirming a tightly bound complex	AfroDB (ZINC)	[164]
(-)-pipoxide (AfroDB)	Aldose reductase	Molecular docking SBVS, ADMET, MD, MM/PBSA	Similar to (+)-pipoxide: docking score -44.77 to-51.46 kJ mol^-1^, strong ligand-key AR residue interactions; ADMET and MD showed good pharmacological profile and stable complex		
Namidine A (AfroDB)	Aldose reductase	Molecular docking SBVS, ADMET, MD, MM/PBSA	Docking scores in the range of -44.77 to -51.46 kJ mol^-1^; key binding interactions and ADMET profiles indicate potential as AR inhibitor; MD confirms the stability of the complex.		
1,6-di-O-p-hydroxybenzoyl-β-D- -glucopyranoside (AfroDB)	Aldose reductase	Molecular docking SBVS, ADMET, MD, MM/PBSA	Docking scores are also between -44.77 to -51.46 kJ mol^-1^; forms several strong hydrogen bonds with active residues of AR; ADMET is positive (favourable pharmacological profile); MD supports a stable complex		
Neocryptotanshinone (TCMID)	PTP1B (protein tyrosine phosphatase 1B)	Molecular docking TCMID library	One of 180 TCMID compounds, showing the best score in PTP1B active site; proposed as a major competitive inhibitor of PTP1B. (Tested *In Vivo*: decreased blood glucose in db/db mice)	TCMID	[165]
Nimbaflavone (IMPPAT)	MAPK1, PI3K (signalling pathway of T2DM)	Direct molecular docking (IMPPAT)	Strong binding to key T2DM proteins: scores of -36.40 kJ mol^-1^ (MAPK1) and -40.17 kJ mol^-1^ (PI3K), the highest among phytohaemo constituents; supporting the role of flavonoids as antidiabetics via regulating the MAPK/PI3K-Akt pathway	IMPPAT, Dr. Duke’s	[166]

The data assembled in the table speak powerfully to the need for researchers to cast a wide net when searching for starting candidates. Resources such as AfroDB and IMPPAT compile plant-derived compounds from African and Indian folk medicine, instantly expanding the chemical inventory under consideration. A gratifying number of these compounds display multitarget behaviour, which has begun to attract attention for the treatment of multifaceted diabetes. Taken as a whole, Table 6 illustrates how combining *in silico* docking studies with public-domain chemical collections can streamline lead discovery, save laboratory time, and conserve research budgets before anyone steps into a wet-bench assay.

## Integration of *in silico* with other approaches (*in vitro* and *in vivo*)

### In silico - in vitro - in vivo relationship: multi-stage validation

Stages in the process of discovering antidiabetic drugs include doing *in silico*, *in vitro* and *in vivo* analyses. *in silico* methods include computer simulations such as molecular docking and molecular dynamics, which predict the interactions of bioactive compounds with diabetes-related proteins. *In silico* results are later verified *in vitro*, for example, through enzyme assays or cell cultures, after which they are tested *in vivo* on animal models to assess physiological and pharmacokinetic effects. That kind of integrated approach has already been used; for example, Shahzad *et al.* [157] employed a comprehensive research strategy integrating *in vitro*, *in silico* and *in vivo* studies of extracts from Cicer arietinum and Hordeum vulgare. In general, the combination of these three methods enables efficient screening of drug candidates through simulations to narrow the pool, while capturing biological and metabolic parameters that are manifest only in living systems.

In silico simulation is essential for molecular docking of amylase, glucosidase, PTP1B, and other targets. It can determine which compounds have the highest binding affinity. Further testing is performed *in vitro* to quantify effects such as IC_50_ value and cellular impact. The hypoglycemic effect is then validated *in vivo*. As an example, Bukhari *et al.* [167] predicted strong butine binding to NF-κB and caspase-3 proteins with docking scores of -30.96 and -27.20 kJ mol^-1^. Experimentally demonstrated, butin significantly restored biochemical parameters and altered pancreatic morphology in diabetic rats, suggesting structural recovery. Conformational changes alongside his retention and recovery suggest enhanced residual aid in regression towards normal physiology post-biofeedback-driven therapy. If simulations differ from experimental data, this suggests the need for model adjustments that incorporate factors such as bioavailability and metabolism. Thus, results from *in vitro*/*in vivo* experiments not only refine computational simulations but also reconfigure calibrations, thereby substantially enhancing accuracy and ultimately strengthening the models relied upon.

Table 7 lists the antidiabetic agents that have been scrutinized both *in silico* and subsequently verified *via in vitro or*, in some instances, *in vivo* experimentation. This two-step methodology links digital predictions to observable biological behaviours, thereby supporting claims about the therapeutic potential of each compound and its underlying biochemical mechanisms. The roster comprises standalone plant metabolites, crude herbal preparations, entirely synthetic molecules, and hybrid entities forged by laboratory manipulation of natural scaffolds. Molecular targets span a broad range, from carbohydrate-modifying enzymes such as α-amylase and α-glucosidase to phosphatase PTP1B, peptidase DPP-IV, glucose transporter GLUT-4, and energy-sensing AMPK. Inflammatory checkpoints, including NF-κB and caspase-3, also play a prominent role in the treatment of hyperglycemia and its associated inflammatory responses.

**Table 7.** *In silico* research added with *in vitro* and/or *in vivo* methods. The addition of these two methods can provide a correlation between research conducted *in silico*. This correlation can validate the *in silico* method that has been conducted, or to determine the molecular mechanism of the performance of the antidiabetic compounds used

Compound	Molecule target	Methods			Results	Ref.
		*In silico*	*In vitro*	*In vivo*		
Medicagol (phytochemical *C. arietinum*, natural compound) and *Hordeum vulgare* methanol extract (natural compound)	α-amylase, α-glucosidase	Molecular Docking, ADMET profiling, MD Simulation	α-amylase, α-glucosidase inhibition	Oral glucose tolerance test, pancreatic and intestinal glucoside activity, G6PD activity, glycogen estimation, biochemical analysis, oxidative stress biomarkers	Docking showed high affinity; IC50 α-amylase: 55.08 μg mL^-1^ (*C. arietinum*), 115.8 μg mL^-1^ (*H. vulgare*); α-glucosidase: 100.25 μg mL^-1^, 216.25 μg mL^-1^. *in vivo* (STZ mice): ↓ blood glucose, ↓ lipids, ↑ antioxidants (SOD, CAT, GSH)	[157]
Butin (flavonoid, natural compound)	NF-κB, caspase-3, insulin	Molecular docking, MD Simulation	N/A	Hb1Ac estimation, blood glucose estimation, insulin level measurement, glycogen estimation, creatinine estimation, lipid profile, AST and ALT estimation, biochemical parameters estimation, antioxidant activity (SOD, GSH and CAT) assay, MDA estimation, Pro‑inflammatory cytokine determination, estimation of caspase‑3, histopathological study	Docking: -30.96 (NF-κB), -27.20 (caspase-3), -34.31 (insulin) kJ mol^-1^; *in vivo* restoration of metabolic parameters, pancreatic structure, and reduction of inflammation	[167]
Caffeic acid (phenol, natural compound)	PTP1B	Molecular Docking	N/A	Glucose level measurement, morphometric assessment of drosophila larvae, crawling assay, survival assay, gene expression analysis	PTP1B docking affinity is stronger than reference; 500 μM dose decreases hyperglycemic drosophila hemolymph glucose, increases larval survival and movement	[168]
*Merremia tridentata* extract (natural compound)	α-amylase, α-glucosidase	Molecular docking	N/A	Biochemical analysis (blood glucose level, TG, HDL, LDL, TC, VLDL and hepatic glycogen level	Docking: cynaroside as a strong inhibitor; IC_50_ *in vitro *α-amylase 1.61 mg mL^-1^, α-glucosidase 0.24 mg mL^-1^; *in vivo* vercomes hyperglycemia better than glibenclamide	[169]
*Crotalaria quinquefolia* extract (natural compound)	α-amylase, α-glucosidase	Molecular docking, MD, QSAR	Antioxidant activity (DPPH, FRAP, reducing power), antidiabetic activity (α-glucosidase and α-amylase inhibition assay)	Oral glucose tolerance test, diabetogenic effect, toxicity test	Docking: high affinity for myricetin, quercetin, rutin, kaempferol; IC_50_ *in vitro* α-amylase 12.8 μg mL^-1^, α-glucosidase 36.3 μg mL^-1^; *in vivo* decreased glucose 18.9 % (rat OGTT)	[170]
synthetic 2-aminobenzothiazole derivative (synthetic)	PPARγ	ADMET properties, molecular docking	N/A	Acute oral toxicity, β-cell function and insulin resistance assessment, insulin level assessment, quantifycation of glucose, HbA1c, TG, T-Cho, HDL-C, LDL-C, activity of the enzymes ALT/GPT, AST/GOT and GGT	Docking: Δ*G* ≈ -32.64 (3b) and -35.15 (4y) kJ mol^-1^ on PPARγ; *in vivo* (T2D mice) pioglitazone equivalent dose, blood glucose <200 mg dL^-1^, improved lipid profile	[171]
4-nitrophenoxysobutyrate (synthetic)	PPAR-γ and GLUT-4	Molecular docking, MD simulation	GLUT-4 and PPAR quantification	Blood glucose estimation	Nitro derivative (comp.2) of dual active PPAR-γ (↑PPARγ/GLUT-4 expression), ↓blood glucose in hyperglycemic mice; high docking score (π-π binding at Gln-286).	[141]
NP-analogous compounds (natural/ synthetic)	AMPK	Protein modelling, database generation, molecular docking	Kinase assay	N/A	Two candidate NP-analogs activated AMPK (AMPK activity increases of ~1.65× and 1.58× at 30 μM)	[172]
1-O-ethyl-β-D- -(6→3′)-glucopyranosyluridine (natural compound)	AMPK	Molecular docking, MD simulations, ADMET prediction	AMPK activation assay	N/A	This uridine derivative compound increased AMPK phosphorylation in a dose-responsive manner in HepG2 cells (↑ p-AMPK/AMPK ratio)	[173]
*Gymnema sylvestre* extract (natural compound)	α-amylase, α-glucosidase	Molecular docking, MD simulations	DPPH assay, FRAP assay, α-amylase, α-glucosidase inhibition	N/A	Methanol extract strongly inhibited carbohydrate enzymes (IC50 α-amylase 57.4 μg mL^-1^; α-glucosidase 218.5 μg mL^-1^). Docking: compound 6 binds strongly (score ) ~ -10.0/-9.1 kJ mol^-1^)	[174]
*Solanum lasiocarpum* extract (natural compound)	α-amylase, α-glucosidase, DPP-IV	Molecular XP docking, ADMET analysis, MD simulations	α-amylase inhibition assay, α-glucosidase inhibition assay, DPP-IV inhibition assay, cytotoxic activity on cell lines, SRB assay, glucose absorption assay using 2-NBDG, kinetic study of 2-NBDG uptake, DPP-IV inhibition assay	N/A	IC50 enzyme inhibition: ~2.12 mg mL^-1^ (α-amylase, α-glucosidase, DPP-IV); extract increased cellular glucose uptake; docking (XP/MM-GBSA) identified >10 α-amylase and DPP-IV inhibitory compounds	[175]
New (synthetic) benzylidene-2,4- -thiazolidinedione	PPAR-γ	Molecular docking, pharmacophore model analysis, MD simulations	N/A	Acute oral toxicity, oral glucose tolerance, body weight effect identification,	Compounds 5d and 5e showed antihyperglycemic effects in mice (STZ) equivalent to rosiglitazone; docking: 5c,5d scores -42.26 and -41.84 kJ mol^-1^ (higher than native partial agonist -40.00 kJ mol^-1^)	[18]
Arylbenzofuran (*Morus mesozygia*, natural compound)	α-glucosidase, DPP-IV	Molecular docking, drug-likeness study	α-amylase inhibition assay, α-glucosidase inhibition assay, DPP-IV inhibition assay	N/A	Compounds 1-3: IC50 α-glucosidase 16.9; 16.6; 40.9 μM (10 to 30× more potent than acarbose). α-amylase was not affected. DPP-IV: moderate inhibition (compound1 ~15 % at 100 μM). Docking: compound 2 (Moracin P) affinity -39.75 kJ mol^-1^ (H-bond)	[143]
7-Fluorochromone-thiomaacetylbazone (synthetic)	α-glucosidase	Molecular docking, ADMET analysis, MD simulations, QSAR modelling	α-glucosidase inhibition assay	N/A	Chromone-thiosemicarbazone derivatives strongly inhibit α-glucosidase (highest IC_50_ of 6.40 μM for compd. 3k with acarbose ~870 μM). Docking/MDS: π-π bonds and H-bonds in the active site enhance the activity	[147]

Molecular docking and molecular dynamics simulations form the first phase of this investigation, providing computational scaffolding before the protocols are shifted to bench-level assays. In-house enzymatic activity assays, paired with longitudinal measurements of serum glucose, lipid fractions, and oxidative stress markers in rodent models, establish a direct link between in silico predictions and biological outcomes. Medicagol, butin and caffeic acid emerged from these workflows as notable glucose-lowering agents, each enhancing antioxidant defences.

Synthetic scaffolds, such as the 2-aminobenzothiazole series and 4-nitrophenoxyisobutyrate derivatives, act as PPARγ agonists, signalling an increase in GLUT4 at the tissue periphery. Plant-derived mixtures, such as chickpea (*Cicer arietinum*), spotted rattlepod (*Crotalaria quinquefolia*), and gymnema (*Gymnema sylvestre*), block carbohydrate-hydrolysing enzymes with an inhibition profile that, in some cases, outpaces acarbose. The IC_50_ values against target enzymes, along with the OGTT results, lend quantitative support to these qualitative observations. Collectively, the dataset underscores how integrating computational prediction with hands-on biology can accelerate the search for safer and more effective small-molecule agents for diabetes.

### Future directions: bioinformatics, big data and personalized medicine

Modern bioinformatics approaches have integrated various biological datasets, including “big data” from multi-omics and electronic health records, to develop more precise and personalized anti-diabetes therapeutics. Conceptually, such integration shifts from the paradigm of “one drug fits all” to a computed analysis of the molecular profile of the patient, such as genome, transcriptome, proteome, metabolome, and even microbiome, to stratify pathways of specific disease processes, predict drug response, and tailor-make treatment to minimize adverse effects. A recent review published in Biomedicines highlights that the pharmacomultiomics strategy identifies gene variants, such as SLCO1B1 and CYP2C9, that alter the pharmacotherapy of metformin, sulfonylureas, and glinides, thereby enabling individualized dosing [176].

The development of artificial intelligence (AI) and machine learning (ML) has accelerated this pace. Current neural network models can mine millions of entries from ZINC-PubChem, predict ligand affinities, simulate binding dynamics, and extrapolate systemic effects through metabolic pathway networks. Farnoud *et al.* [177] highlighted that AI-trained pipelines on *in silico* - *in vitro* datasets could reduce preclinical costs by more than 50 % while improving the hit rate for anti-diabetic drug candidates. On the other hand, the pharmacogenomic framework based on big data from hospitals enables the HLA variant detection that would trigger the rare side effects, lactic acidosis associated with metformin and then directly integrated into the physician's e-prescribing systems, which are predicted to become a global standard [178].

Applied research has already demonstrated significant impact. An *in silico* study by Shapiro *et al.* [179] mapped 11 million ZINC compounds to the crystal structure of DPP-IV using tiered QSAR-based AI and validated the top 5 hits *in vitro*; two of them, ZINC000003015356 and 15786214, inhibited DPP-IV activity in Huh T1 cells over 80 % at 10μM and showed minimal toxicity to pancreatic β cells. Another example, Kirchweger *et al.* [172], focusing on temporal context: expression of the phosphorylated AMPK (proteome) and lipid profiles (metabolome) were analysed alongside docking small synthetic ligands; the best AMPK activator increased hepatocyte glucose response over 1 to 6 times compared to control.

Looking ahead, three distinct trends are emerging. (1) The testing of digital twins of diabetes patients, simulated replicas of a person’s anatomy, which hyper-realistically emulate their physiology in real time, is commencing within AI simulations for insulin dose adjustment [180]. (2) Privacy concerns in multinational collaborations are addressed through federated learning, where drug-prediction models are trained in various institutions without necessitating raw data sharing. (3) New possibilities for correlations between glycemic control and integrated lifestyle factors, including therapy efficacy, are unlocked with the merger of continuous glucose monitoring paired with wearables for activity, glucose, and streamlined through cloud big data platforms, expert opinions on drug discovery review 2022 [177].

Still, the same challenges persist: heterogeneity of data formats, population bias, high computation requirements, and a regulatory framework for AI models. However, the consensus from literature from the last seven years emphasizes that a synergy of bioinformatics, AI/big data, and the principles of personalized medicine will form the cornerstone for the next generation of discovery and designing anti-diabetic therapy, shifting research from just “prediction of protein docking” to a comprehensive understanding of the patient’s system biology and evidence-based computational therapy optimization.

## Conclusion

The *in silico* Anti-diabetes compound prototyping approach, using domain predictive modelling of molecular docking, molecular dynamics simulation, QSAR, and ADMET prediction, has demonstrated the capability to screen millions of molecular compounds at low cost while accurately predicting affinity and stability of ligand-protein binding, associating deconvoluted chemical features with biological activity, and eliminating problematic candidates early on. The literature ranges from 2018 to 2025 reviewed demonstrates that several natural compounds, such as aril-benzofuran, mangiferin, and gluggusterone E, as well as novel synthetic analogues such as bis-Schiff base, thioskarbazon, and coumarin-chalcone hybrids exhibited binding energies and IC_50_ values exceeding those of standard inhibitors, acarbose and vildagliptin. Virtual hits targeting α-glucosidase, DPP-IV, PPARγ, TGR5, GK, PTP1B, SGLT2, and GLUT4 have been validated for MD block stability (50 to 150 ns) and, in many cases, have demonstrated activity in cell or animal models. Reported integration of *in silico*, *in vitro* and *in vivo* phasing showed a more than 50 % reduction in preclinical costs while increasing hit-rate for drug candidates. Regardless, unrestricted empirical docking scores based on protonation assumptions, QSAR/ADMET domains of validity, and data heterogeneity demanding layered biological validation-model refinement have posed a challenge, emphasising the need for ongoing model improvement.

In this case, the article proposes a few directions for further research. First, apply artificial intelligence and big data using multimodal deep learning and federated learning to enable collaborative analysis of inter-institutional pharmacological datasets without compromising confidentiality. Second, create a “digital twin” of the patient by integrating genomic and metabolomic profiling with pharmacokinetic simulations to support personalized therapy. Third, expand the use of dynamic pharmacophores extracted from MD trajectories and standardize free-energy rescoring to reduce false negatives in virtual screening. Fourth, enriched ADMET models with experimental data (Caco-2 permeability, hERG, hepatotoxicity) so that their accuracy approaches industrial standards and included PBPK simulations starting from hit-to-lead to assess hypoglycemia risk and tissue distribution. Fifth, continuing the exploration of new targeted or selective modulation, such as PPARγ partial agonists, GK allosteric activators, TGR5 gut-specific agonists, or dual combinations of α-glucosidase and PTP1B to enhance efficacy and minimize side effects. Lastly, a uniform open-access repository containing ligands, docking protocols, MD trajectories, and IC_50_ data is needed to improve reproducibility and cross-study benchmarking. Achieving these objectives will enable the scientific community to advance from predicting protein interactions to designing holistic, personalized, and translatable-to-clinic therapies for diabetes.
